# Symmetry breaking in the embryonic skin triggers directional and sequential plumage patterning

**DOI:** 10.1371/journal.pbio.3000448

**Published:** 2019-10-02

**Authors:** Richard Bailleul, Camille Curantz, Carole Desmarquet-Trin Dinh, Magdalena Hidalgo, Jonathan Touboul, Marie Manceau

**Affiliations:** 1 Center for Interdisciplinary Research in Biology, CNRS UMR7241, INSERM U1050, Collège de France, Paris Sciences et Lettres University, Paris, France; 2 Sorbonne Université, UPMC Univ Paris 06, Laboratoire Jacques-Louis Lions, Paris, France; 3 Department of Mathematics and Volen National Center for Complex Systems, Brandeis University, Waltham, Massachusetts, United States of America; Cancer Research UK London Research Laboratories, UNITED KINGDOM

## Abstract

The development of an organism involves the formation of patterns from initially homogeneous surfaces in a reproducible manner. Simulations of various theoretical models recapitulate final states of natural patterns, yet drawing testable hypotheses from those often remains difficult. Consequently, little is known about pattern-forming events. Here, we surveyed plumage patterns and their emergence in Galliformes, ratites, passerines, and penguins, together representing the three major taxa of the avian phylogeny, and built a unified model that not only reproduces final patterns but also intrinsically generates shared and varying directionality, sequence, and duration of patterning. We used in vivo and ex vivo experiments to test its parameter-based predictions. We showed that directional and sequential pattern progression depends on a species-specific prepattern: an initial break in surface symmetry launches a travelling front of sharply defined, oriented domains with self-organising capacity. This front propagates through the timely transfer of increased cell density mediated by cell proliferation, which controls overall patterning duration. These results show that universal mechanisms combining prepatterning and self-organisation govern the timely emergence of the plumage pattern in birds.

## Abstract 2

A survey of plumage patterns and their emergence in Galliformes, ratites, passerines, and penguins shows that their formation depends on a species-specific prepattern in the embryo and demonstrates that universal mechanisms govern the timely emergence of natural patterns in birds.

## Introduction

The diverse shapes and motifs that adorn animals have been a long-standing interest of theoreticians and developmental biologists: how can patterns arise from homogeneous structures during the development of an organism in an often highly organised and reproducible manner? On the one hand, numerous modelling studies, frequently assuming a chemical basis for pattern-forming factors (for review [1,2]) but also recently integrating cellular and mechanochemical processes (for review [3,4]), led to the theorisation of self-organising dynamics to explain the emergence of many patterns. However, a single final pattern can often be reproduced by a variety of models [4,5]. In addition, each of these models (both in their equations and stationary solutions) potentially describes various developmental mechanisms. Choosing and building models that not only accurately anticipate patterns but also guide relevant tests of in vivo patterning mechanisms thus often remains challenging. On the other hand, genetic screens and expression analyses of developmental factors—sometimes guided by modelling—have identified candidate molecules and cellular events putatively involved in pattern formation in vivo [1,2,6,7]. However, biological interpretation is often limited by the difficulty to link a given pattern to prior molecular gradients and/or cell behaviours occurring in the absence of spatial reference in the a priori naïve, unpatterned tissue.

**Fig 1:**
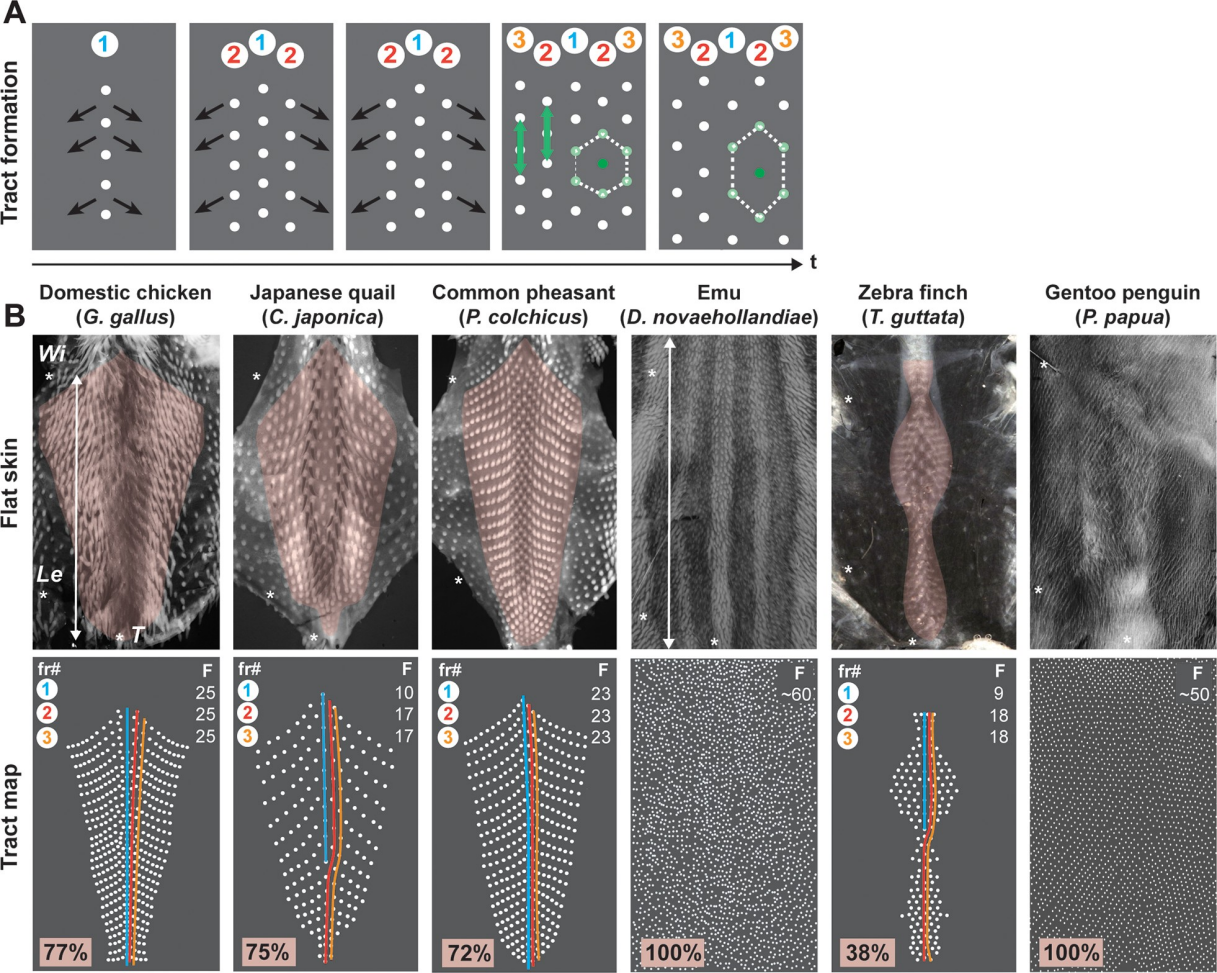
Dorsal tracts vary in shape, size, and geometry between birds. (A) During development, the dorsal tract appears in a medial–to–lateral bidirectional wave (black arrows) of fr (fr#1–3 are schematically represented), resulting in a micropattern in which each feather follicle (one of them is shown in dark green) is surrounded by a regular hexagon of neighbours (light green). This pattern later distorts along the anteroposterior axis (green arrows). (B) Flat preparations of dorsal skins (upper panels) and their corresponding schematic map (bottom panels) show that completed dorsal tracts (in pink) vary in size and shape between the domestic chicken (in which it covers around 77% of the dorsum surface; *n* = 2), the Japanese quail *Coturnix japonica* (75%; *n* = 3), the common pheasant *Phasianus colchicus* (72%; *n* = 3), the emu *Dromaius novaehollandiae* (100%; *n* = 3), the zebra finch *Taeniopygia guttata* (38%; *n* = 2), and the gentoo penguin (100%; *n* = 1). In the first three species and the zebra finch, feather follicles (in white) organise in longitudinal rows (fr; circled numbers) that extend from the neck to the tail and contain a reproducible number of feathers (‘F’, counted from wings to tails as shown by white arrows): F = 25, 10, 23, and 9 for fr#1 (in blue; note that in the Japanese quail and the zebra finch, this row is shorter because it is composed of late–developing feathers [12,22]), and F = 25, 17, 23, and 18 for fr#2 and fr#3 (in orange and red). In the emu at E26 and the gentoo penguin at around E25, we quantified means of F ≈ 60 and F ≈ 50 feathers as quantified along tract length, respectively. Stars show the position of wings (‘Wi’), legs (‘Le’), and tails (‘T’). E, embryonic day; fr, feather row; t, time.

The plumage pattern is one of the few emblematic systems in studies of pattern formation and evolution in which the modelling–experimentation gap has been successfully bridged [5,8]. In birds, feathers are implanted in so-called tracts (or ‘pterylae’) separated by glabrous areas. The spatial distribution of tracts at the scale of the whole body (i.e., macropattern) is broadly conserved, all birds having capital (head), humeral/alar (wings), dorsal, ventral, femoral/crural (legs), and caudal (tail) tracts [9,10]. However, their shape and size, as well as the geometrical arrangement of feathers within tracts (i.e., micropattern), vary between bird groups (as formerly studied in the zoological field of pterylography; [11,12]). Work performed in the domestic chicken *Gallus gallus* showed that the patterning of feather tracts involves surface dynamics occurring in the developing skin tissue. In this species, the early mesodermal layer first directs the formation of competent epidermal areas (or feather fields). Within each feather field, longitudinal rows of feather follicles individualise in a medial-to-lateral “wave” of differentiation to form a regular dotted pattern, which later becomes distorted to give rise to a reproducible geometry in which each feather is surrounded by an elongated hexagonal array of neighbours [10] (Fig 1A). Feather arrangement is thus a complex yet ordered motif that results from the timely orchestration of patterning events in the developing skin including (1) local individualisation of shapes (here, follicles) from an initially homogeneous feather field and (2) the directional and gradual progression of this differentiation process.

Efforts to understand plumage patterning have largely focused on the control of feather follicle individualisation. Computer simulations of self-organising models (e.g., reaction–diffusion and chemotaxis alone or in combination) can give rise to regularly spaced dots reminiscent of the chicken feather motif [13,14] (or other cutaneous structures in vertebrates [15,16]), which suggests that the formation of feather follicle arrays results from a self-organisation of the developing skin. The nature of self-organising positional factors raises much interest, as recent studies have evidenced a coupling between molecular and mechanically driven, cellular dynamics. Several candidate proteins of the bone morphogenetic protein (BMP), fibroblast growth factor (FGF), and Wnt signalling pathways have been identified based on their local expression and diffusive properties and/or because perturbing their expression/activity modifies the local arrangement and differentiation of feather follicles [13,14,17,18]. Such molecular activity may rely on spontaneous changes in cellular organisation: the nuclear translocation of *β*-catenin in epidermal cells (an event marking early stages of feather follicle differentiation [19]) is caused by a local aggregation of dermal cells as the result of their contractile properties [20]. Conversely, cellular dynamics can also depend on molecular activity: FGF signalling drives dermis aggregation, consequentially compressing the epidermis, which in turn intensifies FGF expression in a positive feedback loop [21].

However, neither self-organising models nor the abovementioned molecular/cellular processes explain the directional and progressive aspects of plumage pattern formation. Consistent with the medio-lateral appearance of feather rows, the differentiation of the skin tissue is characterised by a dorsoventral gradient of cell density [9]. This gradient has been shown to precede a wave of Eda signalling that sets the cell density threshold required for the occurrence of feather follicle individualisation [21]. However, the mechanisms that trigger such a signalling wave are not known. In addition, the events that mediate its propagation within the feather field and control the orientation and temporal dynamics of this process such that it creates a stepwise sequence of longitudinal row formation have not been identified. Thus, to what extent the control of patterning in time shapes the organisation of the avian skin in space and constrains its evolution remains an unresolved question.

Here, we tackled this challenge by performing an extended survey of tract pattern formation in Galliformes, passerines, ratites, and penguins, thereby identifying common and varying attributes of its temporal emergence. We built a mathematical model that intrinsically reproduces both these dynamics and their variation. We achieved so by combining parameters of reaction–diffusion and chemotaxis with logistic cell proliferation; when applied on initial conditions measured in the naïve skin tissue of each species, our unified model recapitulated species-specific patterning dynamics without forcing onto an extrinsic mathematical wave. We then verified predictions by showing in vivo and ex vivo that the two key elements driving spatiotemporal dynamics are (1) an early symmetry-breaking event occurring prior to follicle individualisation and oriented along the anteroposterior axis and (2) the nonhomogeneous proliferation of cells, behaving as predicted by the logistic source and created by the limited capacity of the tissue to support cell density. These phenomena trigger the progressive appearance of discrete longitudinal domains in a travelling wave. The area of each segment, modulated by the prepattern, accommodates the self-organisation of a given number of follicles, which gives rise to the row-by-row patterning sequence in species with sharp enough initial conditions. By characterising skin architecture through time, we demonstrated that a lateral transfer of increased cell density dictates the directional propagation of longitudinal segments. Finally, we showed that colchicine-based inhibition of cell proliferation on cultured skin explants slows down patterning in line with model simulations, demonstrating that cell proliferation sets the overall duration of tract completion.

## Results

### Dorsal feather tracts vary in macro- and micropattern between birds

To facilitate the identification of events involved in the temporal control of plumage pattern formation, we first described common points and differences in its final organisation by performing a comparative survey of completed dorsal tracts. The domestic chicken historically served as model: consistent with previous work [9], we observed that when completed (at embryonic day [E]11), its dorsal tract covers approximately 77% of the skin surface and displays an orderly arrangement in which adjacent longitudinal rows (i.e., feather rows, or “fr” in figures) contain a reproducible number of feather follicles (F = 25 as quantified between wings and tail in medial rows) and form ‘chevrons’ along the dorsoventral axis. For comparison, we chose two close relatives in the Galliformes bird order (which is part of the monophyletic group Galloanserae), namely the Japanese quail *C*. *japonica* and the common pheasant *P*. *colchicus*, for which we previously described feather distribution [22]. In Galliformes, overall tract size (about 75% and 72% of dorsum surface, respectively), shape, and geometry are conserved, whereas the number of feathers per row is species-specific: F = 17 in the quail (except in fr#1, known to develop later, in which F = 10 [17]) and F = 23 in the pheasant. By contrast, we found that in the emu *D*. *novaehollandiae*, which is a flightless ratite of the derived monophyletic group of paleognaths, feathers are arranged across the whole dorsum in a dense and irregular pattern (F ≈ 60, as quantified along lines drawn from wings to tail). Because Galleoanserae and Palaeognathae represent two ancient, species-poor branches of the bird phylogeny, we extended our survey to include two species of Neoaves, third and major avian taxon comprising 95% of all living bird species [23]. In the zebra finch *T*. *guttata*, a passerine bird in which tracts had been previously thoroughly described [10], the dorsal tract encompasses a relatively thinner skin region (about 38% of the dorsum surface) compared to Galliformes. It displays a different shape with an enlargement at the level of hind limbs (so-called saddle), with fewer and shorter rows that are less strictly arranged (9 < F < 18 in the medial rows). Finally, in the gentoo penguin *Pygoscelis papua*, which is an aquatic Neoaves bird devoid of flight abilities—much like the emu—we found that feathers densely cover the whole dorsal region (F ≈ 50; Fig 1B). Together, these observations show that independently of dorsum size, both the macropattern (relative tract surface and shape) and the micropattern (number and geometry of feather follicles) vary between species. Drastically opposite plumage patterns within taxa (such as for the Neoaves zebra finch and gentoo penguin) suggest that variation relies on changes in otherwise broadly conserved mechanisms.

### Spatiotemporal dynamics of tract formation vary between species

To link variation in tract patterns to temporal dynamics of their emergence, we characterised the spatial organisation of dorsal feather fields prior to, and during, the appearance of feather follicles. To do so, we chose to assess the expression of *β-catenin*, which marks differentiating follicles [19], but also whose signalling acts upstream of FGF (which plays a role in feather follicle individualisation) and the receptor of Eda (EDAR, which marks the travelling wave of follicle differentiation [21]). To avoid bias caused by visually assessing potentially species-specific gene expression to determine the presence/absence of a forming follicle, we developed an automatic algorithm complementing manual counts that consistently sorts and quantifies the number of follicles in microscopy images of each species (S1 Fig). We found that in all Galliformes and in the zebra finch, *β-catenin* initially forms one visible medial (B1) and two lateral (B2) longitudinal bands, in which follicle individualisation gradually takes place, starting at the posterior end of the band and progressing anteriorly. Adjacent feather rows form according to the same steps and sequentially, in a row-by-row wave that travels ventrally and stops at the limit of the feather field, with most follicles in a given row having formed prior to the appearance of the next *β-catenin*-expressing band. Additional feather rows also fill up the dorsal-most space between B2 bands. Follicles reach the limit of the tract in about 2–3 days, irrespective of the duration of the whole development (varying from 14 days in the zebra finch to 16–22 days in Galliformes). Thus, in these four bird species, tract patterning is characterised by broadly conserved medial-to-lateral bidirectionality, sequence (i.e., row-by-row dynamics and individualisation prior to next row), and duration. These spatiotemporal attributes may therefore be controlled by shared developmental mechanisms. We, however, observed subtle variation in the initial *β-catenin* pattern: although lateral bands B2 are similar in all species, the medial band B1 extends from the interlimb region to the tail in the chicken, whereas in the Japanese quail it is reduced to the posterior region, in the common pheasant it is diffuse, and in the zebra finch it is fused to lateral bands B2, forming a Y-shape. This profile defined the species-specific location of first-individualising follicles, suggesting a link between the spatial organisation of the feather field prior to the onset of the differentiation wave and the timely dynamics of its progression.

**Fig 2:**
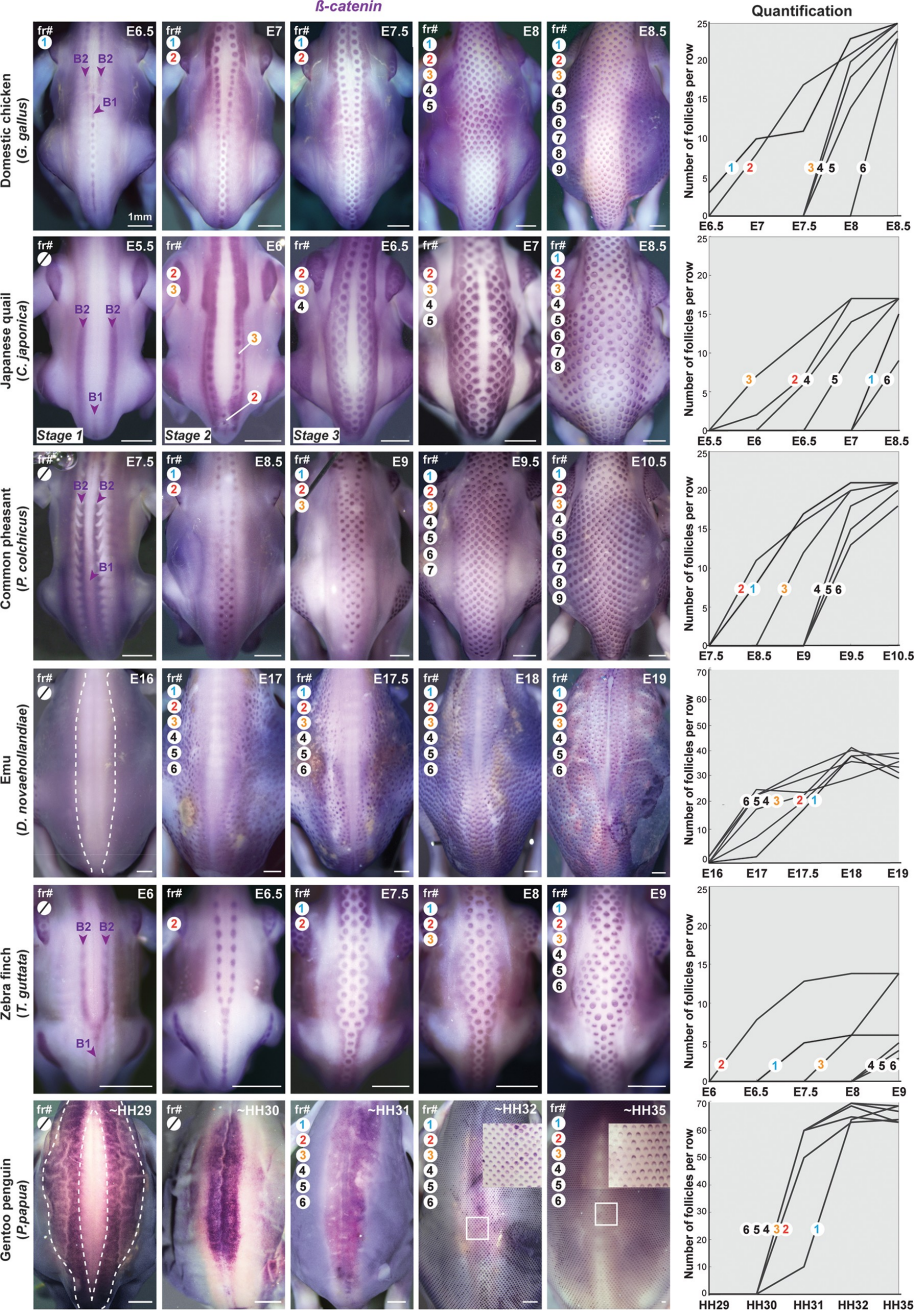
Dorsal tract formation varies in directionality, sequentiality, and duration. In situ hybridisations revealing *β–catenin* transcripts (in purple, left panels) mark the dynamic appearance of fr at equivalent developmental stages in each species (*n* values are listed in S1 Table). In the domestic chicken, the Japanese quail, the common pheasant, and the zebra finch, the first fr arise from three medial bands: one posteriorly located (B1) and two anteriorly located (B2). Lateral rows then appear one by one in a front that progresses ventrally and stops at the limit of the dorsal tract. In the emu, *β–catenin* is first seen throughout the dorsum but in a thick medial region (white dotted line); follicles then individualise progressively at random locations. In the gentoo penguin, two wide *β–catenin*–expressing lateral surfaces (white dotted line) precede a switch to the central region. Only then do follicles appear rapidly throughout both herein defined surfaces, creating a regular squared geometry (see magnifications, white squares). Automatic quantifications of F per row (right panels) show that fr#1–6 appear in a sequential manner in Galliformes and the zebra finch: follicle individualisation occurs in a given row when the preceding row is near completed (with the exception of fr#1, where it has species–specific timing, and fr#2 in the Japanese quail, where it is first restricted posteriorly). In contrast, along virtual longitudinal lines in the emu and the gentoo penguin (see S1 Fig), follicles individualise simultaneously with no apparent spatial order. The colonisation of the feather field lasts 2–3 days in Galliformes, and the zebra finch and is comparably faster in the emu and the gentoo penguin. HH stage 1, stage 2, and stage 3 are defined in Fig 5 and Fig 6; scale bars: 1 mm. E, embryonic day; fr, feather row; HH, estimated Hamburger and Hamilton stage.

In contrast with the first four species, *β-catenin* expression initially marks the whole dorsal region except its central part in the emu. Within this primary *β-catenin*-expressing surface, follicle individualisation occurs randomly in space and time over the course of roughly 1 day (emus develop in 60 days). Similarly, in gentoo penguins, *β-catenin* marks large regions of the dorsal feather field: first detected in two thick lateral bands, it rapidly switches to the complementary central region and later covers the whole tract. It thereby defines two successive early patterning surfaces, throughout which follicle individualisation rapidly takes place (gentoo penguins develop in 35 days). Contrary to the emu, penguin follicles readily appear in a highly regular pattern, forming a unique squared geometry (Fig 2). This difference aside, follicle patterning occurs in both flightless species through dynamics that neither are directional nor have the clear, slower-paced row-by-row sequence seen in Galliformes and the zebra finch, and in a comparably shorter duration. This may be due to entirely different patterning mechanisms specific to ratites and penguins or to drastic changes in dynamics of tract establishment that occur independently of follicle individualisation.

### A unified mathematical model reproduces both shared and varying directionality, sequentiality, and duration of tract formation

To guide the identification of events controlling the timely emergence of tract patterns, we built a model that intrinsically generates both the shared and varying attributes of directionality, sequence, and duration we observed in the different species. This model combines several spatiotemporal phenomena, mathematically described as partial differential equations and previously shown to generate spatial dotted arrays.

We first included reaction–diffusion models, first theorised by Alan Turing [24] and involving the diffusion of at least one local self-activating factor *u* and its longer-range inhibitor *v* [24–27]. Such models have been widely used to explain the establishment of natural patterns and the effect of several candidate pathways such as FGF and BMP signalling [1,2]. We simulated a range of reaction–diffusion equations for various interaction functions *f* and *g* on homogeneous surfaces:

$$\begin{array}{c} \partial_{t}u=D_{u}\Delta u+f(u,v)\\ \partial_{t}v=D_{v}\Delta v+g(u,v) \end{array}$$

Consistent with previous studies, we found that when initiated on small random fluctuations, such models can generate dotted patterns but do not reproduce directionality or row-by-row sequence observed experimentally (S2A Fig). Other self-organisation models create motifs by combining locally evolving cell density *n* (according to a proliferation rate *p*) with chemotaxis, the process of cell migration in response to a chemoattractant *u* generated by the cells (at a rate *α*_*u*_), and also subject to diffusion (diffusivity *D*_*u*_) and degradation (rate *δ*_*u*_) [28]:

$$\begin{array}{c} \partial_{t}n=D_{n}\Delta n-\nabla .(\kappa n\nabla u)+p(n)\\ \partial_{t}u=D_{u}\Delta u+\alpha_{u}n-\delta_{u}u \end{array}$$

Such models are appealing to explain feather tract formation because they can represent epidermal/dermal interactions and the gradient of cell density occurring during the differentiation of the feather field [28,29], two processes tightly linked to the presence of a travelling wave [21]. However, although chemotaxis models are sufficient to produce dotted arrays [28], they too failed to intrinsically produce directionality or sequentiality in spot formation (S2B Fig). Because we observed that *β-catenin* initially forms medial bands in Galliformes and the zebra finch, we then ran simulations of self-organising models on frames containing a longitudinal line. We found that in this case, models produce repeated longitudinal bands within which spots individualise simultaneously (i.e., without row-by-row sequence), mimicking tract formation observed in skin areas of the emu and the gentoo penguin initially marked by *β-catenin* (S3 Fig). Thus, pattern directionality can be generated by combining self-organisation with spatial heterogeneities oriented along the medial axis and present in the skin prior to follicle individualisation.

However, such combination is not sufficient to produce the row-by-row pattern sequence seen in Galliformes and the zebra finch, which argues for the involvement of other events orchestrating the dynamics of tract establishment in these species. Temporal sequences of pattern formation have been obtained when chemotaxis-driven cellular distribution and/or reaction–diffusion were applied to mathematical, medial-to-lateral priming waves: condensed structures then form in a chicken-like sequence of directionality and speed related to the properties of the wave [13,14,21]. Similar modelling in other study systems (e.g., sensory organs in *Drosophila*) also yielded sequential patterning [30]. We thus aimed at producing sequentiality to follicle individualisation using similar models but devoid of extrinsic forcing. To do so, we described the evolution of local cell density *n*(*t*,*x*) according to two mechanisms. First, cell density changes because of diffusion, and chemotaxis changes towards instantaneous concentrations of an activator chemoattractant *u*(*x*,*t*) depending on both its autocatalytic production by cells and the concentration of its repressor *v*(*x*,*t*). Second, cell density is modulated by intrinsic logistic proliferation accounting for a division rate *α*_*n*_ at low cell population levels and a carrying capacity of the tissue *β*_*n*_, density above which proliferation stops. This term is inspired from a widely studied mathematical framework that serves as a classical tool to generate spreading events in theoretical studies of population behaviour and infectious diseases propagation [31] and efficiently captures temporal dynamics of cell migration in vitro [32]. The resulting unified model thus contains three partial differential equations as follows:

$$\begin{array}{c} \partial_{t}n=D_{n}\Delta n-\nabla .(\kappa n\nabla u)+\alpha_{n}n(1-\frac{n}{\beta_{n}})\\ \partial_{t}u=D_{u}\Delta u+\frac{\alpha_{u}n(1+{\omega u}^{2})}{\left(\beta_{u}^{2}+u^{2})(1+v\right)}-\delta_{u}u\\ \partial_{t}v=D_{v}\Delta v+\alpha_{v}nu^{2}-\delta_{v}v \end{array}$$ (1)

*D*_*n*_, *D*_*u*_, and *D*_*v*_ parameterise the diffusion of cells, attractor, and repressor, respectively, whereas *κ* accounts for the sensitivity to chemotaxis; *α*_*u*_ (*α*_*v*_) is the production rate of the attractor (repressor) by the cells; *β*_*u*_ and *ω* respectively quantify the saturation threshold and autocatalysis sensitivity of the activator; and *δ*_*u*_ (*δ*_*v*_) is the degradation rate.

**Fig 3:**
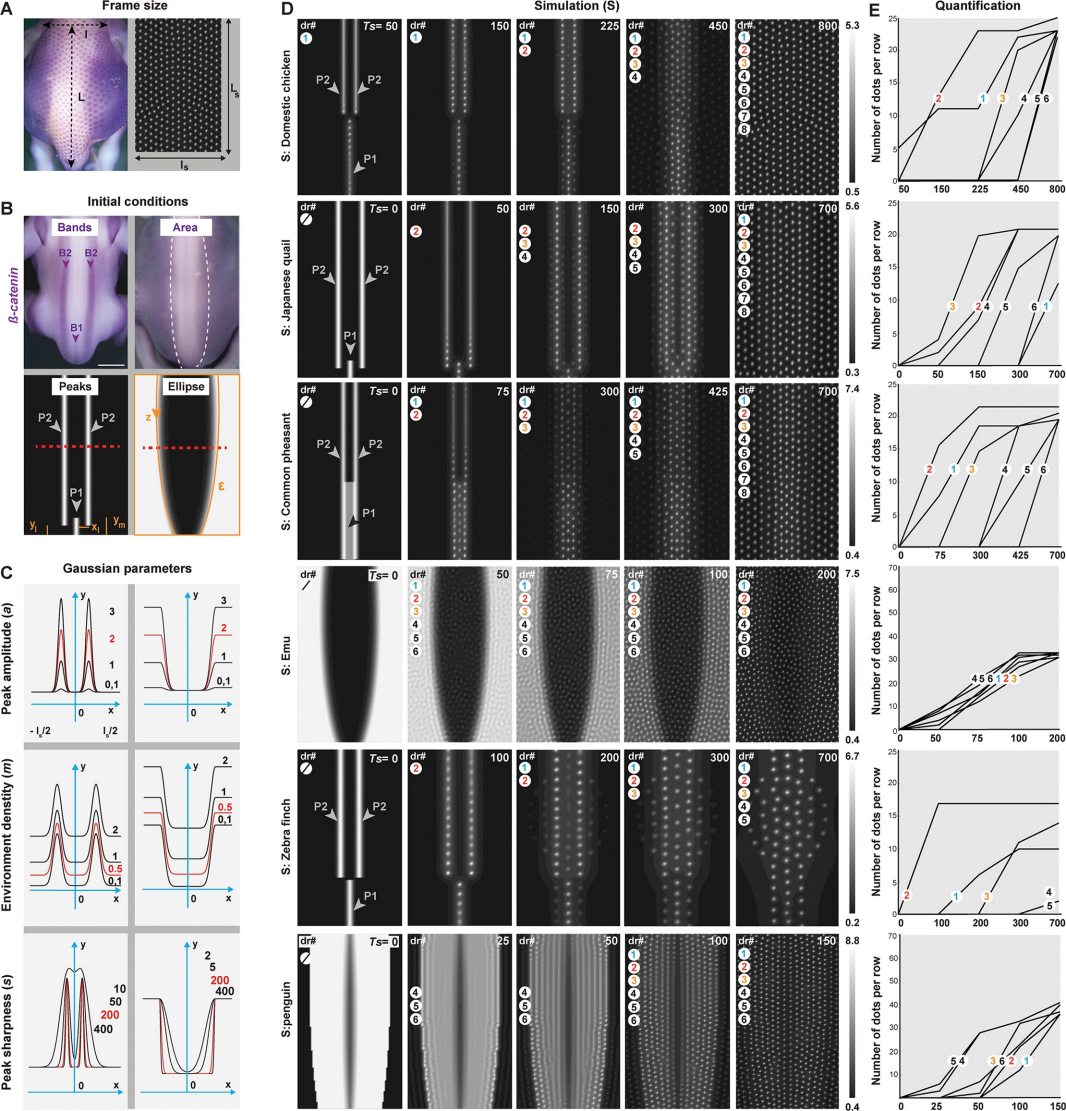
Simulations of the unified model reproduce spatiotemporal attributes. (A) The size of each simulation frame was defined so that its abscissa l_s_ corresponds to the distance between wings l and its ordinate L_s_ to the distance between wings and tails L (as shown here on a domestic chicken embryo at E8.5) and reported to obtain F quantified when the model reaches an equilibrium. The obtained ratios for l/l_s_ and L/L_s_ were then used to define the size of simulation frames for the other five species (all values are shown in S2 Table). (B) Initial conditions for P1 and P2 corresponding to measurements of the central location of B1 and B2 *β–catenin* bands relative to body landmarks and the ratios reported in the Japanese quail are defined by *x*_*l*_ and *y*_*m*_, *y*_*l*_, respectively (left panels). The elliptic surface *ε* representing the *β–catenin–*expressing surface of the emu is parametrised by z (right panels). (C) Schematics showing the effect of variation in amplitude *a* (upper panels), density outside of peaks *m* (middle panels), and sharpness **s** (bottom panels), compared to reference values (in red), on gaussian and elliptic curves corresponding to Eqs 2 and 3, respectively, at the level of red dotted lines in (B). Axes are shown in blue; 0 represents the dorsal midline; **l**_**s**_: frame width; **x**, abscissa; **y**, ordinate. (D) In silico simulations (‘S’) of the unified model were run on frames corresponding to the relative size of the dorsal tract in each species as shown in (A) and species–specific numerical initial conditions as shown in (B) with reference gaussian and elliptic parameters shown in (C). At various intermediate simulation times (*Ts*; indicated in white, upper right corners), dots individualise in rows. The formation of dr occurs in a bidirectional and sequential manner in the Galliformes and zebra finch simulations and simultaneously throughout the frame in the emu and gentoo penguin simulations (each mimicking in vivo species–specific dynamics). Simulations stabilise with motifs corresponding to the final macro–and micropatterns observed in respective species. For each species–specific simulation, colour bars indicate minimal–to–maximal *n* values in a black–to–white gradient. (E) Automatic quantifications of dots at various simulation times confirm that the unified model reproduces both shared directionality/sequentiality in tract formation. dr, dotted rows; E, embryonic day.

We first performed a stability analysis of the model around spatially uniform solutions and theoretically identified pattern-forming instabilities (S4A Fig and S1 Text), recovered when simulating the system from small random initial conditions (i.e., without axial initial conditions). As expected, it allowed the formation of regularly spaced dots that appear throughout the simulation surface in a timely fashion (S4B Fig). Interestingly, the stability analysis showed that all elements of the model take part in pattern formation. In particular, reaction–diffusion or chemotaxis terms are crucial to pattern formation (see Eqs 7 and 10 in S1 Text). Because we showed that initial axial conditions provide directionality to pattern formation, we next added species-specific spatial heterogeneities to numeric simulation frames by adapting their sizes to that of tract size/shape in each bird (Fig 3A) and building geometrical initial conditions corresponding to the initial expression pattern of *β-catenin* in each species. To achieve the latter, we used for Galliformes and the zebra finch three gaussian equations restricted along the axes so that their peaks correspond to the measured central location of B1 and B2 *β-catenin* bands:

$$n_{0}\left(x,y\right)=m+ae^{-s_{m}x^{2}}\left(y<y_{m}\right)+a(e^{-s_{l}{\left(x-x_{l}\right)}^{2}}+e^{-s_{l}{\left(x+x_{l}\right)}^{2}})\left(y>y_{l}\right)$$ (2)

For emus and gentoo penguins, we respectively used one (as shown below) or two (see Material and methods) elliptic surfaces *ε* to approximate the shape of initial areas marked with *β-catenin* (Fig 3B):

$$n_{0}(x,y)=(m+a)1_{\varepsilon }(x,y)+m1_{\varepsilon^{c}}(x,y)+a1_{\varepsilon^{c}}(x,y)(e^{-s_{\varepsilon }{\left(x-z\right)}^{2}}+e^{-s_{\varepsilon }{\left(x+z\right)}^{2}})$$ (3)

In these equations, *x* and *y* are variable coordinates; *x*_*l*_, *y*_*m*_, and *y*_*l*_ correspond to measures relative to body landmarks that position the medial (P1) and lateral (P2) peaks; **1**_*ε*_ and $1_{\varepsilon^{c}}$ are indicator functions that define inner and outer regions of elliptic surfaces *ε*; and *z*(*y*) is a variable value parametrising the elliptic curve (see Materials and methods). The amplitude and sharpness of peaks and elliptic areas, respectively, are represented by *a*, *s*_*m*_, *s*_*l*_, and *s*_*ε*_, and *m* represents a minimal density throughout the simulation frame (Fig 3C). We found that simulations of the model with a unique set of reference parameters chosen from the stability analysis for their pattern-forming abilities (S4 Fig) and conserved minimal density but species-specific peak conditions allow the formation of individualised dots in a bidirectional and typical row-by-row sequence as well as in the dorsal space between initial lines, requiring similar times of simulation *Ts* to stabilise in a final pattern for Galliformes and the zebra finch (*Ts* = 700/800) and in a spatially random and faster manner in the emu and the gentoo penguin (*Ts* = 200/150; Fig 3D and 3E). Thus, combining self-organisation (which controls follicle individualisation) and logistic proliferation with a species-specific spatial organisation of the yet-unpatterned skin (as marked by the expression of *β-catenin*) accurately anticipates patterning directionality, sequence, and overall duration. Together, the incremental building of a unified model reproduced shared and varying spatiotemporal attributes of tract pattern emergence, allowing us to predict that they rely on a prepattern upon which spontaneous mechanisms act to produce individualised follicles.

### Symmetry breaking triggers directional patterning from initial conditions

**Fig 4:**
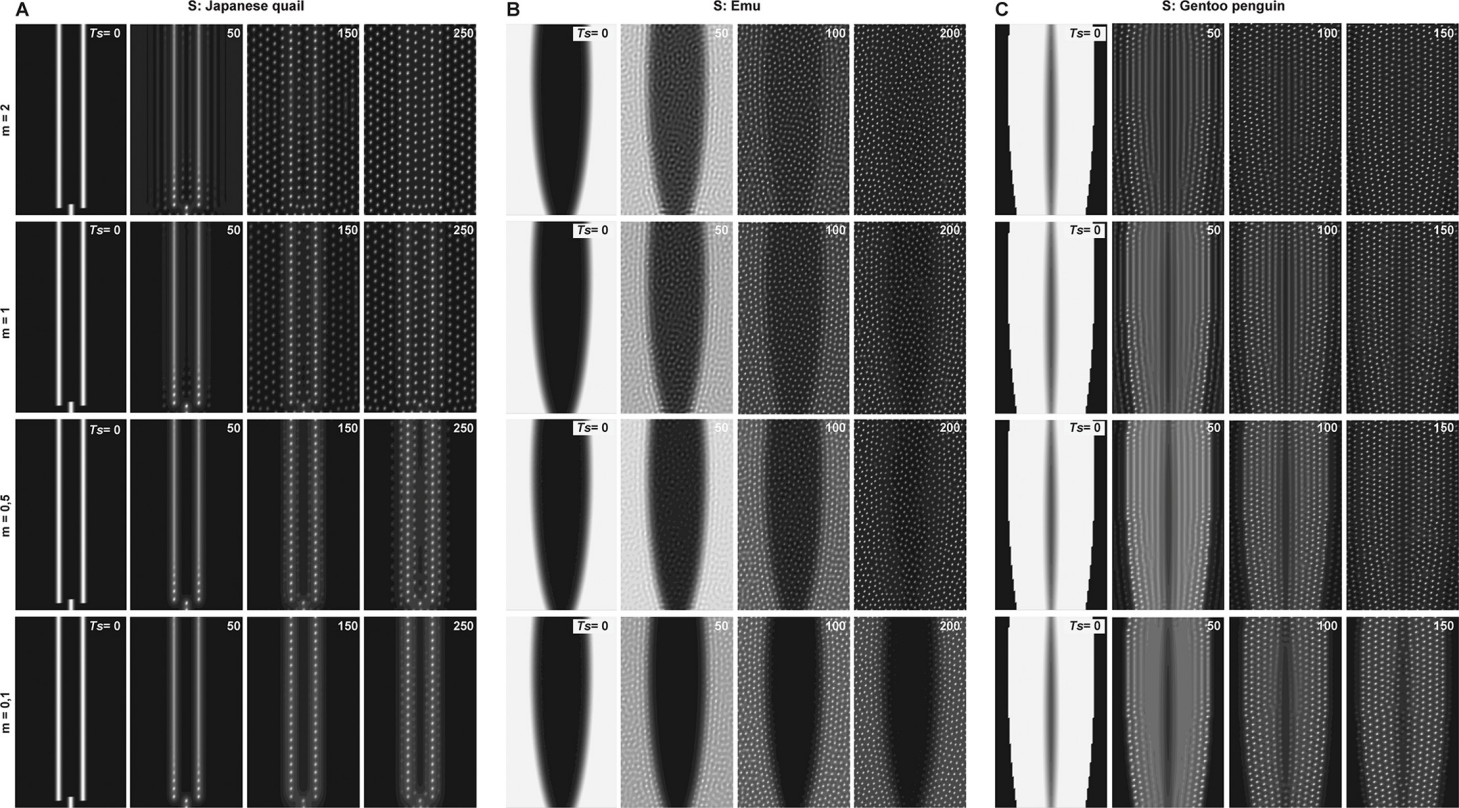
Varying minimal density modifies patterning sequentiality. (A) Simulations of the unified model with initial conditions corresponding to the Japanese quail show that dots form in a row–by–row sequence when the minimal density outside of gaussian peaks *m* is low but form simultaneously when *m* > 0.5. Simulations with initial conditions corresponding to the emu (B) or the to the gentoo penguin (C) show that dots consistently form simultaneously throughout the frame when *m* increases, whereas low values of *m* cause a delay in dot formation outside of elliptic surfaces, consistent with in vivo dynamics observed in both species. Ts, simulation time.

To characterise the prepattern, we generated model-based predictions by varying parameters defining gaussian peaks (corresponding to longitudinal bands or areas in vivo) and their surrounding environment (corresponding to the feather field). We used simulations with Japanese quail–like initial conditions, which are the simplest profile of primary bands, or emu- and gentoo penguin–like initial conditions, which are definite areas. We found in all cases that varying *a*, the parameter representing the amount of causal factor in longitudinal bands or areas, does not affect the final pattern or its dynamics of formation (except slightly with emu-like conditions at the highest *a* value; S5 Fig). Thus, patterning occurs independently of the initial number of cells and/or molecules responsible for follicle differentiation within bands. It is, however, impacted by the number of factors outside of peaks, described here by *m*: with Japanese quail–like conditions, proper row-by-row sequence is lost when *m* reaches a high threshold. Strikingly, however, we found that sequential patterning occurs with low *m* values (close to *m* = 0; Fig 4A). Thus, conserving low minimal density of cellular/molecular factors outside of domains spatially restricted along the medial axis is an initial symmetry-breaking event that endows only the regions within these domains with the capacity to form patterns (neighbouring regions having close to basal density unable to generate patterns). Even when small, this break in symmetry acts as a trigger, sufficient to launch the medial-to-lateral patterning wave in which cells near longitudinal peaks progressively transmit the pattern-forming capacity to neighbouring regions. By contrast, we found that with emu- and gentoo penguin–like conditions, dots form without clear sequence within initial surfaces independently of *m* values, suggesting that it is the profile of initial conditions rather than their amplitude that controls pattern sequentiality. In these cases, however, *m* influences the duration of tract completion: lowering *m* with emu or gentoo penguin–like initial conditions delays pattern formation in central regions not primarily defined by *β-catenin* expression, more accurately reproducing in vivo dynamics (Fig 4B and 4C). This is consistent with recent work showing that optimal density provides patterning competence to the feather field [21] and suggests that pattern differences in these species can be due to comparably lower amounts of cellular/molecular factors in the feather field environment surrounding initial conditions.

### Initial conditions control the patterning sequence

To understand what controls the timely sequence of patterning, we assessed the role of self-organising events (as they have been previously involved in intrinsic properties of patterning waves [13,14]). To do so, we varied all self-organisation parameters on fixed Japanese quail–like initial conditions. We observed changes in the size and spacing of dots when, all parameters otherwise equal, we modified the activator or repressor diffusion *D*_*u*_ or *D*_*v*_ (S6 Fig); the chemotaxis sensitivity κ (S7 Fig); the strength of molecular interactions *α*_*u*_, *α*_*v*_, *β*_*u*_, and *ω* (S8 Fig); or the degradation rate *δ*_*u*_, *δ*_*v*_ (S9 Fig). All tested values preserved row-by-row dynamics of dot emergence—except for the most extreme. The latter often caused an absence of individualisation, in all cases matching exactly patterning criteria resulting from the stability analysis, even when two parameters are modified (S6–S9 Figs). Thus, together, simulations suggest that self-organisation controls follicle size and spacing consistent with previous findings [5,14,33] but have no impact on the onset of the row-by-row wave nor are responsible for its sequential aspect. Interestingly, simulations defined by species-specific frames and *β-catenin* profiles yielded a number of dots per row similar to that observed for follicles in Galliformes and the zebra finch and lower to that of the emu and the gentoo penguin (compare with Figs 2 and 3). An appealing explanation is that parameters of self-organising events that control the individualisation of follicles in vivo are constrained by parameters defining the prepattern.

**Fig 5:**
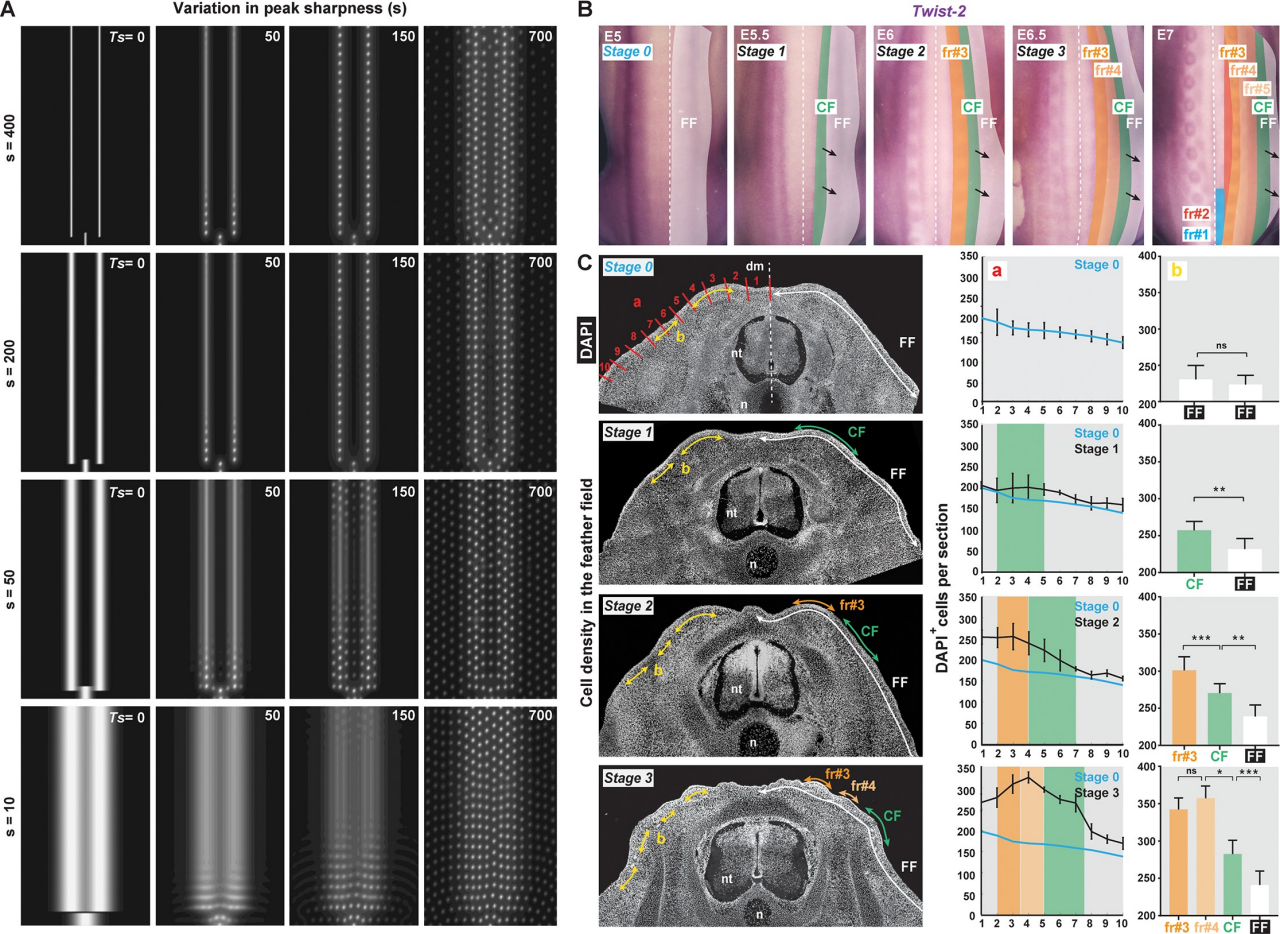
Sequentiality results from the progressive appearance of longitudinal domains. (A) Simulations of the unified model with initial conditions corresponding to the Japanese quail show that increasing peak width *s* leads to a loss of row–by–row sequence, whereas decreasing *s* has no effect on pattern emergence. (B) In situ hybridisations revealing *Twist–2* transcripts (in purple) from E5 (stage 0) to E7 mark the progressive appearance of longitudinal domains in a CF (black arrows; CF in green) within the FF (in white) and later forming fr (i.e., at that level in the Japanese quail, fr#3 and fr#4 that form first, shown in shades of orange, followed by fr#2, fr#1, fr#5, respectively, in red, blue, and light orange). (C) Left panels: local changes in cell density can be observed on transverse sections of Japanese quail embryos stained with DAPI to mark nuclei (in white) prior to *β–catenin* expression (stage 0), at initial conditions of *β–catenin* expression (stage 1), and during the individualisation of fr#3 and fr#4 (stages 2 and 3). Middle panels: quantifications of skin cells within 10 sections corresponding to the red bars in **a** in the left panel show that cell density is homogeneous at stage 0 (Friedman test; *p* = 0.26) and gradually increases in a travelling medial–to–lateral wave (results obtained at stage 0 are shown in blue in all other graphs for comparison). Right panels: quantifications of skin cells within sections of the same length positioned according to *β–catenin* expression (see S10 Fig, S1 Data, and Materials and methods) and corresponding to yellow arrows in **b** in the left panel show that from stage 1, cell density significantly increases between segments that express *β–catenin* and those positioned immediately laterally in the FF (Student *t* test; *p* = 0.19 at stage 0; *p* = 0.001 at stage 1; *p* = 0.0002 and 0.0039 at stage 2; *p* = 0.36, 0.017, and 0.0008 at stage 3). This reflects the dynamic appearance of competent domains that accommodate the formation of one follicle (i.e., fr#3 at stage 2 and fr#4 at stage 3). CF, competence front; E, embryonic day; FF, feather field; fr, feather row; n, notochord; ns, nonsignificant; nt, neural tube; Ts, simulation time.

We next tested the role of initial conditions: we performed complementary simulations in which self-organisation parameters are all maintained but the width of Japanese quail–like primary longitudinal bands containing causal factors is modified. We found that when we increased peak width (by decreasing *s*), several dot rows could form simultaneously within the herein produced surface, mimicking patterning in the emu and the gentoo penguin, whereas a decrease (by increasing *s*) did not modify the resulting pattern or its sequential formation (Fig 5A). We therefore hypothesised that follicle individualisation occurs within discrete competent domains whose area is set by initial conditions and hosts the formation of a definite number of rows. Consistent with this model-based prediction, we found that in the Japanese quail, the mesoderm and follicle marker *Twist-2* [34], though unlikely to contribute to the induction of patterning because it is expressed throughout the feather field prior to the “trigger stage” (i.e., corresponding to initial *β-catenin* expression), delineates longitudinal surfaces appearing in the same order as future feather rows (Fig 5B). To characterise these putative domains in vivo, we quantified local cell density by counting DAPI-positive skin cells in epidermal and dermal layers (as both contribute to feather follicle formation and are not separated in the unified model). We used either blind counts throughout the feather field divided in 10 sections of equal lengths (to quantify overall cell density) or counts limited to sections located within and just lateral to *β-catenin*-expressing bands (to compare local cell density in and outside of forming rows; S10 Fig). We found that prior to the trigger stage (stage 0), cell density is slightly higher in the medial part of the dorsum and gradually decreases ventrally, reflecting the previously evidenced gradient [9] but not correlating spatially with observed and simulated Japanese quail initial conditions (which, contrary to the domestic chicken, are positioned laterally to the medial axis). However, at the trigger stage (stage 1), cell density peaks where *β-catenin* expression appears (i.e., at the level of the putative first row), dropping sharply at the boundary with the lateral, *β-catenin*-negative environment. During the formation of the first row (fr#3 and the posterior part of fr#2; stage 2), the amplitude of the peak increases, marking the differentiation of a follicle, as a second region of comparable area with increased cell density appears laterally. The latter also increases and becomes flanked by a third area during the formation of the second row (fr#4; stage 3; Fig 5C and S1 Data). Thus, cell density gradually increases locally, defining successive longitudinal domains that form one follicle row. Together, simulations and experimental data indicate that a travelling front of longitudinal surfaces defined by causal factors propagates such that follicles individualise with or without row-by-row sequence: the latter occurs as domains have a sharp enough spatial profile that self-organising parameters, controlling follicle size and spacing within the domain, authorise the formation of only one feather follicle at a time.

### Cell proliferation controls patterning propagation and the duration of tract formation

**Fig 6:**
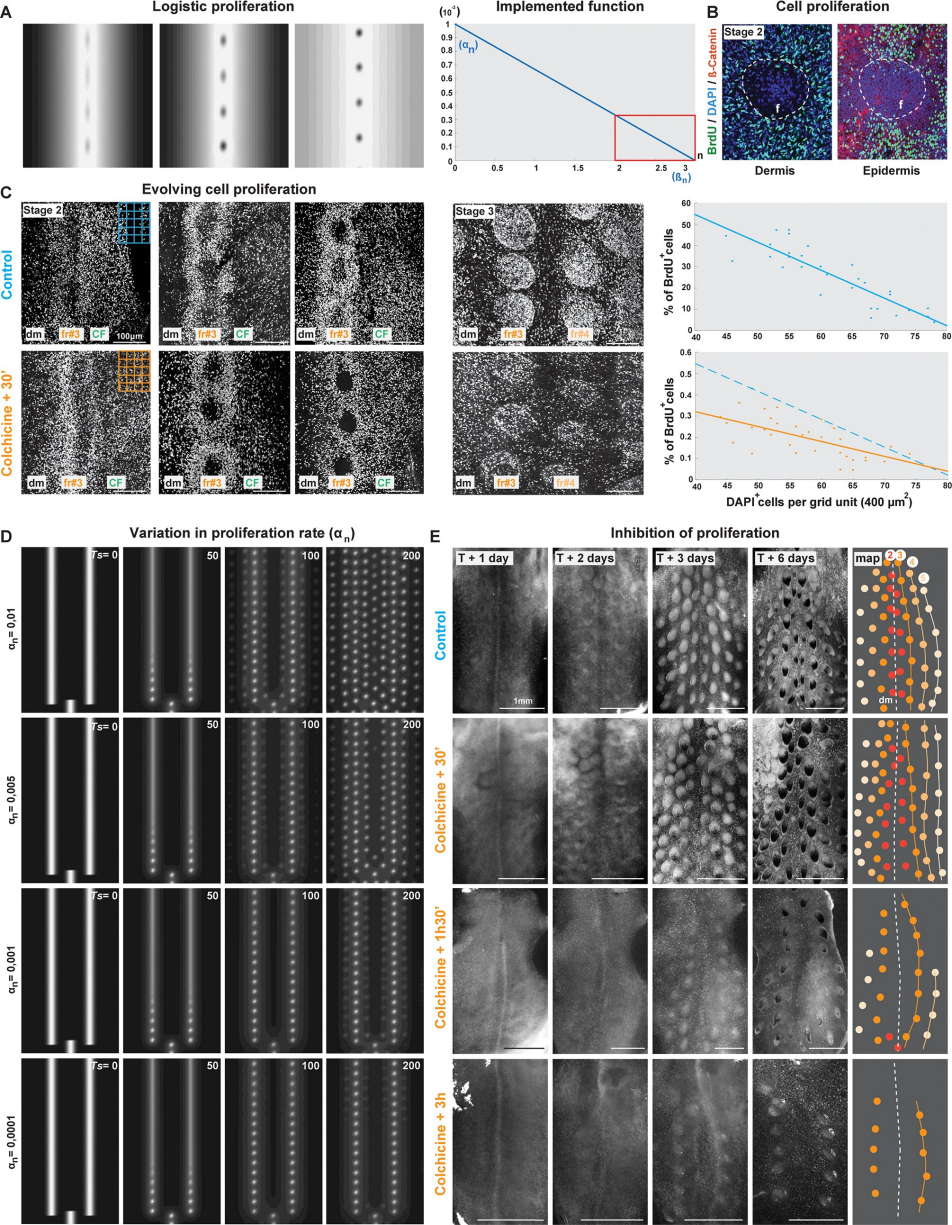
Cell proliferation controls the duration of tract formation. (A) Left panels: simulations of the logistic source on a longitudinal line gradually produce dots whose centre is devoid of proliferation. Right graph: implementation of the logistic term as a function of cell density **n**. (B) Confocal images of forming follicles (‘f’) at stage 2 stained with DAPI (to reveal cell nuclei), *β*–catenin (which marks the epidermis, thereby allowing distinguishing between skin layers), and BrdU (to reveal proliferative cells) show that cell proliferation ceases at the centre of follicles. (C) Left panels: BrdU stains on control (in blue; upper panels) and colchicine–treated (in orange; bottom panels) skin explants of Japanese quail embryos prepared at E6 and fixed at early, intermediate, and late stage 2 (i.e., during the formation of fr#3) or at stage 3 (i.e., when fr#3 and fr#4 are visible) and corresponding to DAPI stains shown for control skins in S13 Fig show that the spatial distribution of proliferative behaviour is conserved in drug–treated skins. Right graphs: quantifications of DAPI^+^/BrdU^+^ cells per surface unit (400–μm^2^ grid such as shown at early stage 2, upper right corner; dot plot; S2 Data) and linear regression analysis of these quantifications (lines) show that the proliferation rate drops with increased cell density (number of DAPI^+^ cells in abscissae) and according to predictions of the logistic source for high *n* values (red square in A) in both control and colchicine–treated explants. (D) Simulations of the unified model with initial conditions corresponding to the Japanese quail show that dot rows form through longer simulation times *Ts* (in white) when the overall proliferation rate *α*_*n*_ decreases. (E) The dynamic appearance of tracts occurs in a progressively slower manner in cultured explants of Japanese quail skins (prepared prior to the differentiation of the feather field) observed from 1 to 6 days (T + 1 to T + 6) after exposure to increasing pulses of colchicine treatment (from 30 minutes to 3 hours), compared to untreated explants (schematics represent fr in the resulting tract with the colour code used previously in Fig 2 and Fig 5). Scale bars, 1 mm. BrdU, 5–Bromo–2′–deoxyuridine; CF, competence front; dm, dorsal midline; E, embryonic day; fr, feather row.

To understand the mechanisms controlling lateral propagation of competent domains, we tested the effect of cell proliferation because (1) our unified model intrinsically generates gradual propagation through a logistic proliferation term and (2) the timely appearance of surfaces with self-organising capacity involves increased cell density. We first simulated the logistic term $\alpha_{n}n\left(1-\frac{n}{\beta_{n}}\right)$ on a longitudinal line and found that it gradually produces ring-shaped structures distributed in a dotted pattern, predicting a cessation of proliferation when density reaches a high threshold (Fig 6A). To validate this prediction, we performed 5–Bromo–2′–deoxyuridine (BrdU) incorporation experiments on skin tissues in the Japanese quail during the formation of the first-formed row (stage 2). We observed for both epidermal and dermal layers a decrease in proliferation at the centre of follicles, where condensation causes higher cell density, and an increase of proliferation in their periphery, where cell density is lower (Fig 6B). Linear regression on quantifications of the proliferation rate (i.e., percentage of BrdU^+^/DAPI^+^ cells in surface units of a grid applied to the whole skin surface; see Materials and methods) demonstrated that it gradually drops with increased cell density (Student *t* test; *p* = 2.8 × 10^−11^). Proliferation stops when the latter reaches a high threshold. When two follicle rows have formed (stage 3), proliferation again becomes visibly homogeneous (Fig 6C and S2 Data). Thus, the logistic term accurately predicts in vivo cell behaviour during follicle differentiation—to our knowledge, a unique empirical validation of this classic mathematical tool.

In light of this observation, we hypothesised that changes in the rate of cell proliferation are also involved earlier than follicle individualisation, in the propagation of the triggered patterning wave. We first assessed proliferation at the trigger stage (stage 1) in the Japanese quail (in which patterning is sequential and lasts 3 days). We found that prior to follicle appearance, proliferation is spatially homogeneous, showing it does not convey symmetry breaking within the feather field (20.3% of all DAPI^+^ cells are BrdU^+^; Friedman test, *p* = 0.26). Comparably in the emu, in which patterning occurs simultaneously and faster, proliferation rate was lower (15.4% of DAPI^+^ cells are BrdU^+^; S11 Fig and S3 Data). These results suggested proliferation rate controls the timely propagation of the patterning sequence. To test this hypothesis, we varied the proliferation rate parameter *α*_*n*_ in a homogeneous manner throughout simulation frames with Japanese quail–like initial conditions. We found that except for most extreme values, varying *α*_*n*_ does not impact follicle size/spacing or the appearance of rows in a sequential fashion; however, reaching final pattern states required longer simulation times as *α*_*n*_ decreased and shorter simulation times as it increased (Fig 6D). We thus tested the effect of the proliferation rate in vivo. To do so, we cultured explants of Japanese quail skin in varying concentrations of colchicine drug, known to inhibit cell proliferation. Low doses did not affect pattern compared to control experiments, whereas high doses had lethal effect (S2 Table). We thus performed pulses of colchicine-mediated inhibition at the highest nonlethal dose. Even with the shortest pulse, this significantly reduced overall cell proliferation during follicle appearance (BrdU^+^ cells are down to 10%–15% of all DAPI^+^ cells, *p* < 0,005, S12 Fig and S4 Data), whereas the spatial distribution of proliferative cells and the formation of individualised follicles remained unchanged compared to control embryos at stage 2. Linear regression analysis showed that colchicine affects the rate of proliferation, whereas the tissue maintains a similar carrying capacity, such as predicted by the logistic source with decreased *α*_*n*_ (Student *t* test; *p* = 2.9 × 10^−5^; Fig 6C). We found that the speed of row formation reduced with longer colchicine pulses (identically to predictions of simulations of the unified model with decreased *α*_*n*_; Fig 6D). As a result, 6 days after treatment, tracts were not complete compared to control experiments (Fig 6E). Together, these results show that the proliferation rate of skin cells mediates the timely propagation of the patterning wave and thus the duration of tract completion.

## Discussion

### The prepattern spatially restricts self-organisation

Altogether, simulations and experimental data allow us to propose a scenario in which in Galliformes and the zebra finch, the tract becomes compartmentalised prior to the individualisation of follicular shapes in periodically arranged, oriented surfaces possessing patterning competence. These longitudinal segments appear in a medial-to-lateral travelling front triggered by a prepattern: in vivo factors are initially spatially restricted such that they create symmetry breaking in the surface field, likely constraining pattern directionality. The propagation of the travelling front is achieved through the lateral transfer of increased cell density controlled by optimal cell proliferation rate, which yields the completion of the patterning process in a timely fashion. Each longitudinal domain self-organises to form one feather follicle row, which produces a row-by-row sequence. Thus, skin pattern formation is a stepwise refinement of space that involves (1) prepatterning, dictating directionality and triggering temporal sequences; (2) cell proliferation, locally controlling patterning propagation and overall duration; and (3) self-organisation, causing follicle individualisation within competent areas. Our work thus shows that periodic patterns are a combination of preexisting and spontaneously generated positional information, highlighting the role of spatial prepatterns in generating temporal dynamics. It also provides an explanation to periodic pattern reproducibility in nature: by constraining self-organisation to definite and oriented compartments, temporal regulation allows the production of repeated motifs in a reproducible manner over large and developing surfaces.

### A framework to identify preexisting positional information

A next challenge will be to pinpoint the nature of the initial symmetry-breaking event, which creates a spatial configuration of the skin surface we modelled by initial conditions n0 in our unified model. Our work thus sets the stage for the identification of such molecular or cellular factors. First, it provides testable hypotheses on their origin (i.e., axial landmarks, such as the neural tube, notochord, or somite) and, hence, their nature: good candidates may comprise neural crest cells, whose migratory paths trigger tissue differentiation [35], or Wnt proteins, which diffuse from the neural tube and somites [36] and activate the expression of *β-catenin* [21]. Second, it informs on the profile of causal factors. By varying parameters of the gaussian peaks defining initial conditions in simulation surfaces, we showed that minor differences in the number of causal factors between the peaks and their surrounding environment are sufficient to launch the patterning wave, provided they are distributed in a sharp enough spatial profile. Consistent with this view, in the emu and the gentoo penguin, shallow initial conditions or high basal levels of causal factors do not sufficiently mark symmetry breaking (consistent with the observation that *β-catenin* is expressed throughout the dorsum surface in these species), and even regions away from the peaks have the ability to self-organise, which results in larger competent areas and a loss of patterning directionality and sequentiality.

Of note, the factors producing initial symmetry breaking may be identical to those contributing to the acquisition of patterning competence. Local differences in cell density, for example, may on the one hand constitute initial spatial heterogeneities triggering patterning, consistent with patterning propagation through the spatial restriction of increased cell density. On the other hand, they may also procure patterning competence, consistent with the observation that emu-like simulations better match the delay in follicle formation observed in the central *β-catenin*-negative region when *m* is decreased and with work from Ho and colleagues showing that follicles form when cell density is above a threshold set by Eda signalling [21].

### Cell proliferation modulates temporal dynamics in the skin

We demonstrated that cell proliferation mediates the lateral propagation of the patterning process to control its overall duration. This temporal attribute is comparable between Galliformes and the zebra finch irrespective of the duration of the whole development and despite variation in the relative size of their tracts. The rate of cell proliferation may thus act as a constraint to the speed of the patterning process and thus create variation in the extent of the plumage pattern in the skin of species with different initial conditions. This raises the possibility that proliferation is a mechanism through which the tract pattern is maintained and evolves. These hypotheses are consistent with the observation that even minor changes in cell proliferation rates through colchicine-mediated inhibition strongly impact temporal patterning dynamics. Further exploring the relationship between cell proliferation and the dynamic distribution of molecular/cellular factors involved in skin differentiation (of which good candidates are molecules of Eda and Wnt signalling pathways) will help gain a better understanding of the mechanisms that control the speed of the patterning wave and/or set the threshold of cell density at which proliferation stops. For example, low values of *m* can also modulate the timing of patterning (Fig 3), raising the possibility that the number of factors in the environment that surround peaks locally influences the rate of cell proliferation. In addition, work in mice showed that hair placode formation—also concomitant with a local decrease in proliferation—is largely driven by differential cell migration [37]. To gain a comprehensive understanding of the cellular events controlling plumage pattern attributes, it will thus be necessary to test the role of cell proliferation at a more local scale, both mathematically through the use of the logistic source and in vivo through real-time tracking of cell division during follicle individualisation.

### On the evolution of tracts and the unique case of penguins

The plumage pattern is characterised by featherless areas that separate tracts and are thought to allow steric space necessary for flight movements [9]. In line with this hypothesis, we showed that feathers cover the whole dorsal skin in a dense pattern in both flightless emus and gentoo penguins, whereas distinctive tracts are observed in closer flying relatives in their respective groups (i.e., the zebra finch for Neoaves and the tinamou for Palaeognathae [21]) as well as in Galliformes (which fly except for adult individuals of domestic breeds of chicken and Japanese quail that have the same tract pattern [22] but are devoid of flight abilities because of increased body weight [38]). The absence of a tract pattern may thus be linked to the loss of flight ability, having evolved repeatedly, and/or through coevolution with changes in wing morphology [39]. Interestingly, we observed surfaces initially devoid of *β-catenin* expression in both emu and gentoo penguin embryos. Whereas in emu it is limited to the central region, which gradually becomes *β-catenin*-positive, in gentoo penguin it is also absent from lateral-most regions, forming two surfaces that can be viewed as deformed or wider Galliformes/zebra finch–like initial conditions. These observations suggest that the tract pattern is an ancestral character, at least to the Neoaves taxon. Reconstructing the evolutionary history of tract pattern formation and flight loss will, however, necessitate a more comprehensive survey of tract patterns that includes other flying and nonflying species in all three avian taxa. In addition, the absence of tracts could be linked to other functions in gentoo penguins such as thermoregulation or water resistance due to adaptation to primary aquatic life and extreme environment, similar to the thickening of their bones or drastic changes in the shape of their feathers. Formal testing of the causal link between tract presence and flight ability would require in-depth, phylogenetic-wide, functional ecological studies.

Differences in initial *β-catenin* surfaces also suggest that similarities in both final plumage patterns and ecologies (i.e., absence of flying ability) have evolved through modifications of distinct developmental mechanisms in emus and gentoo penguins, consistent with the observation that other pattern attributes vary between these species, such as pattern geometry, which is irregular in emus and is the most regular of all observed species with a strict squared geometry in gentoo penguins. Extending the phenotypic survey to other aquatic Neoaves will allow deciphering whether this geometry is a unique feature of penguins. In addition, whereas the pattern of the emu may occur simply through delay of pattern-forming competence [21], the extreme gentoo penguin geometry likely involves new or additional patterning mechanisms, such as other self-organising events and/or changes in the mechanical properties of the developing skin tissue. It will thus be crucial to further explore the mechanisms of follicle individualisation in these emblematic birds.

## Materials and methods

### Ethics statement

All animal work was performed in compliance with regulations for animal use and specimen collection of the French Government and the European Council. Zebra finch breeding colony welfare is guaranteed through regular care and visits; the breeding facility is approved by official and institutional agreement (Direction départementale de la protection des populations and Collège de France, agreement C-75-05-12). Research licenses for gentoo penguin specimen collection have been granted by the Environmental Planning Department of the Falkland Islands Government (R26).

### Embryo sampling and flat skin preparation

Fertilised eggs were collected from a breeding colony at the Collège de France for zebra finches (*T*. *guttata*), from the natural breeding sites of Stevely Bay and Grave Cove in the western part of the Falkland Islands for Gentoo penguins (*P*. *papua*) and from local suppliers for the other species: Les Bruyères élevage for domestic chicken *G*. *gallus*, Cailles de Chanteloup for Japanese quails *C*. *japonica*, Les boix de Vaux for common pheasants *P*. *colchicus*, and l’Emeu d’Uriage and Autruche de Laurette for emus (*D*. *novaehollandiae*). After egg incubation in Brinsea Ovaeasy 190 incubators installed at the laboratory or at the Dunbar Farm (Falkland Islands), embryos were treated in ovo with 9 mg/mL of BrdU (Sigma; BrdU incorporation experiments) and dissected. Flat skins were prepared as described previously [22]. Specimens were fixed in 4% formaldehyde, conditioned and sent back to the laboratory in the case of gentoo penguins, and imaged.

### Expression analyses

In situ hybridisation experiments were performed in each species (*n* is provided in S1 Table) as described previously [40], using antisense riboprobes synthesised from vectors containing 881-bp, 501-bp, and 685-bp fragments, respectively, of Japanese quail, zebra finch, and gentoo penguin coding sequences for *β-catenin* and a 740-bp fragment of Japanese quail coding sequence for *Twist-2*. Digoxigenin-labelled riboprobes were revealed with an anti-digoxigenin-AP antibody (1:2,000, Roche) and an NBT/BCIP (Promega) substrate. Sequences of *β*-*catenin* primers are:

F: AGCTGACTTGATGGAGTTGGA and R: TCGTGATGGCCAAGAATTTC (quail)

F: TAGTTCAGCTTTTAGGCTCAGATG and R: CCTCGACAATTTCTTCCATACG (finch)

F: GAACATGGCAACCCAAGCTG and R: GCCTTCACGGTGATGTGAGA (gentoo penguin). Sequences of *Twist-2* primers are F: AAAGCTCCAGTTCTCCTGTTTC and R: ATGTTGCTTCTCGCTTCTCTG.

Gentoo penguin developmental stages were estimated according to Hamburger and Hamilton classification by morphological comparison with other species.

### Quantifications of tract size and feather follicles/dot number

#### Tract size

Feather-containing surfaces normalised by that of the whole dorsum were measured using Fiji software on pictures of flat skins at developmental stages corresponding to tract completion (E11 for the domestic chicken, *n* = 2; E10 for the Japanese quail, *n* = 3, E12.5 for the common pheasant, *n* = 3, E26 for the emu, *n* = 2, approximately E25 for the gentoo penguin, *n* = 1) and at hatching for the zebra finch (the relative surface of the completed tract being conserved during development, follicles are best visualised at P0; *n* = 2).

#### Follicle/Dot number

To quantify feather follicles or dot number (F) respectively on pictures of flat skins or model simulations in a time-efficient and consistent manner (i.e., across species and at different stages), we developed a custom Matlab program. The algorithm follows three steps: first, a gaussian filter reduces optical noise through image smoothing (Matlab function ‘imfilter’; filters were obtained with the function ‘fspecial’). Second, morphological operations are applied (i.e., closing followed by opening of the image with a disk of radius 1 pixel using functions ‘imopen’ and ‘imclose’; the disk was constructed using the *strel* function). Third, feather follicles/dots are detected as connected components of the image and their properties (centroid, bounding box, surface, etc.) stored. Because background levels on skin images can fluctuate, the algorithm contains a set of linearly spaced thresholds to (1) produce a binary image through a detection threshold of the pretreated image, (2) identify the connected components of the binary image (Matlab function ‘bwconncomp’), and (3) repeat the second step if the maximal value in a given component is above the next threshold.

The resulting feather/dot locations are presented in a Matlab interface (S1 Fig) allowing adjustment of thresholds, filtering of properties, and correction for undetected follicles or false positives (needed for less than 5% of the total follicle number). Locations are processed to identify rows by segmenting the set of feathers/dots according to their location along the x-axis (and corrected by hand in the cases of Japanese quail and zebra finch, for which fr# is shifted laterally, and in the emu, for which counting was performed along six virtual lines). Program code and interface (Dotfinder) are available upon request.

### Modelling

All simulations were performed using FreeFem++ [41] software (specifically designed to compute numerical solutions of partial differential equations) with no flux (Neumann) boundary conditions (i.e., cells or molecules are free to diffuse outside of the tract). Spatial and temporal discretisation parameters were chosen as a compromise between accuracy and efficiency (*n*_*x*_ between 120 and 160, *dt* between 0.01 and 0.1).

#### Size of simulations frames

For comparison of pattern dynamics relative to tract size, the width (l_s_) and length (L_s_) of simulation frames have been set for each species according to developmental landmarks: we measured widths (i.e., l, in mm, distance between wings) and lengths (i.e., L, in mm, distance between tails and a medial point between wings; Fig 3A, S3 Table, and S5 Data). We then adjusted coordinates for the domestic chicken so that the numbers of feather rows (i.e., feather row = 8) and of feathers per row (F = 25) coincide in vivo and in silico. This allowed defining a 0.45 coefficient between L and L_s_ and a 0.7 coefficient between l and l_s_, which were reported to all other species. For better figure readability, simulations were then rescaled to the same dimensions on all plots.

#### Initial conditions

Initial conditions for domestic chicken, Japanese quail, common pheasant, and zebra finch species were implemented with Eq 2 using parameters shown in S4 Table. For the emu, the primary competence domain was approximated by an elliptic surface where **1**_*ε*_ is the indicator function and $z=1.5\sqrt{1-\frac{{\left(y-5\right)}^{2}}{6^{2}}}$ parametrises the ellipse (Eq 3). For the gentoo penguin, the primary competence area was defined as the surface between the two ellipses $\frac{x^{2}}{0.4^{2}}+\frac{{\left(y-3.5\right)}^{2}}{6^{2}}=1$ and $\frac{x^{2}}{1.5^{2}}+\frac{{\left(y-5.3\right)}^{2}}{9^{2}}=1.$ Same initial conditions are implemented on the three variables *n*, *u*, and *v* in each case.

#### Reaction–diffusion models

We tested a large number of reaction–diffusion models (RD1–RD5), as shown in S5 Table. Simulations in S2 Fig were performed using a model recently shown to reproduce a dotted pattern of denticles in sharks [15]:

$$\left\{ \begin{array}{c} \partial_{t}u=D_{u}\Delta u+F(a_{u}u+b_{u}v+c_{u})\\ \partial_{t}v=D_{v}\Delta v+G(a_{v}u+b_{v}v+c_{v}) \end{array}\right.$$

where *F* and *G* are rectifying functions avoiding negative or too large values of the argument: *F*(*x*) = *x* for 0 < 𝑥 < *F*_*max*_, saturating at 0 and *F*_*max*_ outside of this interval, and *G*(*x*) = *x* for 0 < 𝑥 < *G*_*max*_, saturating at 0 and *G*_*max*_ outside of this interval. Simulations were made on a square domain 0 < *x* < 75, 0 < *y* < 150 with parameters *D*_*u*_ = 0.02, *a*_*u*_ = 0.08, *b*_*u*_ = −0.08, *c*_*u*_ = 0.04, *d*_*u*_ = 0.03, *F*_*max*_ = 0.2, *D*_*v*_ = 0.6, *D*_*v*_ = 0.6, *a*_*v*_ = 0.16, *b*_*v*_= 0, *c*_*v*_ = −0.05, *d*_*v*_ = 0.08, *G*_*max*_ = 0.5.

#### Chemotaxis models

We tested a large number of chemotaxis models such as that described in [29].

Simulations in S3 Fig were performed using the model:

$$\left\{ \begin{array}{c} \partial_{t}n=D_{n}\Delta n-\nabla .(\kappa n\nabla u)+\alpha n(1-n/\beta )\\ \partial_{t}u=D_{u}\Delta u+\gamma n-\delta u \end{array}\right.$$

on a square domain 0 < *x* < 30, 0 < *y* < 60 with parameters *D*_*n*_ = 5, *D*_*u*_ = 0.1, *κ* = 5, *α* = 1, *β* = 3, *γ* = 1, *δ* = 1.

#### Unified model

All reference parameters are shown in S6 Table and were largely based on those used in a previous study of chicken-like plumage pattern formation [13]. In the case of the zebra finch, we added a “competence zone” defined by limiting reaction terms within an elliptic surface. We performed a stability analysis as described in S1 Text.

### Skin explants

Skin regions corresponding to putative dorsal tracts were dissected from E6 Japanese quail embryos and placed dermal side down on culture insert membranes (12-wells format, Falcon #353103) over 800 μL DMEM supplemented with 2% FCS and 2% Penicillin/Streptomycin. Stock solutions of colchicine (50 mg/mL in EtOH; Sigma #C9754) were diluted to various concentrations (0.00125–40 mg/mL) in the culture medium to identify the highest nonlethal dose (0.2 mg/mL; S2 Table). Pulse treatments were achieved by washing 0.2 mg/mL colchicine out in successive medium baths after 30 minutes (*n* = 12), 90 minutes (*n* = 7), or 3 hours (*n* = 10), as opposed to untreated, control explants (*n* = 9). Skin explants were incubated at 37°C with a 5% CO_2_ atmosphere (Thermo Scientific Midi 40); medium was changed every 2 days. BrdU assays were performed in explants treated for 30 minutes and cultured to reach stage 2 or 3.

### Immunohistological stains and quantifications

Embryonic specimens were embedded in gelatin/sucrose, sectioned using a CM 3050S cryostat (Leica), treated with HCl 2N for 20 minutes (for BrdU stains), rinsed, and stained using a rat primary antibody directed against BrdU (Abcam; 1:200) or *β*-catenin (Abcam; 1/100) and goat anti-rat Alexa 488 or goat anti-rabbit Alexa 568 secondary antibodies (Abcam; 1:500). Cell nuclei were revealed using DAPI (Southern Biotech). Slides were mounted in Fluoromount (Southern Biotech) prior to imaging. For quantifications of cell density or proliferation, the number of DAPI^+^ or BrdU^+^ cells in both epidermal and dermal layers was counted in 10 sections of confocal images (dividing the dorsal skin from the midline to the ventral limit of the feather field using Fiji software; **a** in Fig 5C) or on portions of 120 μm in length (i.e., shorter than the length covered by visible expression of *β-catenin*, which marks early feather follicle formation) that were positioned within *β-catenin*-expressing areas or lateral to the latter in the feather field, as shown in S10 Fig and in the corresponding DAPI-stained image in **b** of Fig 5C. Homogeneity of cell proliferation along the mediolateral axis at stage 0 in the Japanese quail (*n* = 8) and emu (*n* = 4) was assessed using a Friedman test on 10 sections of identical lengths dividing the whole feather field, and the significance of differences of cell density or proliferation rates from stages 0 to 3 in the Japanese quail was assessed using Student *t* tests on the 120-μm-long sections. For quantifications of cell proliferation rate in control and colchicine-treated explants of Japanese quail skins (3 per condition), BrdU^+^/DAPI^+^ cells were counted using large squares of approximately 150 μm^2^ (to control for overall effect of the drug, as shown in S12 Fig) or a fine-squared grid of 400 μm^2^ (to quantify differences in logistic cell proliferation, as shown in Fig 6C). Linear regression results in a line of equation 1.07–0.0131.x for control conditions (*p* = 2.8 × 10^−11^) and 0.6–0.007.x for colchicine conditions (*p* = 2.9 × 10^−5^). Compared to an equation $\tilde{\alpha_{n}}(1-\frac{n}{\tilde{\beta_{n}}})$ similar to that implemented in the model, this yields an estimated $\tilde{\alpha_{n}}=1,07,$ $\tilde{\beta_{n}}=81.7$ in control conditions and $\tilde{\alpha_{n}}=0.6;$ $\tilde{\beta_{n}}=85.7$ in colchicine conditions.

### Imaging

Flat skins and whole embryos were imaged using an AF-S Micro NIKKOR 60-mm f/2.8G ED macro-lens equipped with a D5300 camera (Nikon) and an MZ FLIII stereomicroscope (Leica) equipped with a DFC 450C camera (Leica). Confocal images were obtained using an inverted SP5 microscope (Leica) with a 40X immersed oil objective.

## Supporting information

S1 Fig Dotfinder and quantification of F in the emu. (A) User interface for the custom Matlab program (Dotfinder) for automatic quantification of feather follicles in pictures of whole embryos stained with β-catenin (a Japanese quail at E7.5 is shown), flat skins, and dots in simulation results. The software allows threshold adjustment of the image, automatic detection, postprocessing of false positives and negatives, and clustering of follicle/dot rows. (B) In the emu, the mean number of feathers per row (F) (i.e., comparable to fr#1–6 in other species) was counted along virtual, equally spaced lines extending from wings to tail (here, at E17). E, embryonic day; fr, feather row.
(TIF)

S2 Fig Simulations of self-organising models on small random fluctuations. Simulations of a (A) reaction–diffusion model [15] or (B) chemotaxis model [29] produce dotted patterns when initiated on small random fluctuations. Ts, simulation time.
(TIF)

S3 Fig Simulations of self-organising models on a longitudinal line. (A) When a reaction–diffusion model [15] is forced onto an initial longitudinal line, it produces stripes that divide into dots to create a dotted motif. (B) When a chemotaxis model [29] is similarly simulated in the same conditions, it produces stripes that simultaneously that divide into dots, starting in the anterior and posterior regions first and travelling towards the centre, resulting in a dotted motif model. Ts, simulation time.
(TIF)

S4 Fig Stability analysis and simulations of the unified model on small random fluctuations. (A) Dispersion relation of the unified model with parameters described in S6 Table and as a function of possible modes of instability **k** (see S1 Text). The curve attains values above 0, showing that the unified model has Turing instability: nonstable modes amplify from small perturbations and can produce patterns with a periodicity related to these modes. (B) Simulations of the unified model (with parameters described in S6 Table) produce dots that appear simultaneously across the whole frame when initiated on small random fluctuations. Ts, simulation time.
(TIF)

S5 Fig Simulations with variation of the *a* parameter. (A) Simulations of the unified model with initial conditions corresponding to the Japanese quail and various amplitude of gaussian peaks *a* (other parameters equal to our reference model; parameter for *a* is *a* = 2) produce dots in a row-by-row sequence with identical times of simulation (*Ts* = 700). Simulations of the unified model with initial conditions corresponding to (B) the emu or (C) the gentoo penguin and various amplitudes of elliptic curves *a* (other parameters identical to our reference model; the reference parameter for *a* is *a* = 2) produce dots simultaneously within the frame with identical times of simulation (*Ts* = 700 and *Ts* = 150, respectively).
(TIF)

S6 Fig Simulations with variation of *D*_*u*_, *D*_*v*_ parameters. (A) Dispersion relation of the unified model with reference parameters (in red [13]) described in S6 Table, extreme low diffusion parameters (in blue) or extreme high diffusion parameters (in yellow), for *D*_*u*_ (left graph) or *D*_*v*_ (middle graph), as a function of possible modes of instability **k** (see S1 Text). No patterns form when the activator diffusion *D*_*u*_ is too high or the inhibitor diffusion *D*_*v*_ is too low. Right graph: depending on values of combined *D*_*u*_ and *D*_*v*_, theoretically derived pattern formation (in white) or homogeneous solutions (in black) occur. (B) Simulations of the unified model with initial conditions corresponding to the Japanese quail, and various diffusivity of the activator *D*_*u*_ (left panels), of the inhibitor *D*_*v*_ (right panels), other parameters otherwise equal (reference parameters are *D*_*u*_ reference = 0.006 and *D*_*v*_ reference = 0.13), produce dots varying in size and spacing but in a row-by-row sequence. Right panels: simulations of the unified model at the equilibrium are consistent with pattern formation when both *D*_*u*_ and *D*_*v*_ vary (i.e., for 9 choices of diffusions; red stars). Ts, simulation time.
(TIF)

S7 Fig Simulations with variation of the chemotaxis parameter *κ*. (A) Left graph: dispersion relations of the unified model with reference parameters (‘ref’, in red) described in S6 Table, extreme low parameters (in blue) or extreme high parameters (in yellow) of *κ* as a function of modes **k** (see S1 Text). No patterns form with extreme low *κ* parameters, but patterns occur with high *κ*. Right graph: depending on values of combined *D*_*u*_ and *κ*, pattern formation occurs (in white) or not (in black). (B) Left panels: simulations of the unified model with initial conditions corresponding to the Japanese quail, and various diffusivity of the sensitivity to chemotaxis *κ* (other parameters equal, references parameters are *κ* ref = 0.00008) produce dots varying in size and spacing but in a row-by-row sequence. Right panels: simulations of the unified model at the equilibrium are consistent with pattern formation when both *D*_*u*_ and *κ* vary (i.e., for 9 choices of diffusions; red stars). Ts, simulation time.
(TIF)

S8 Fig Simulations with variation of the *α*_*u*_, *α*_*v*_, *β*_*u*_, and *ω* parameters. (A) Dispersion relations of the unified model with references parameters (‘ref’, in red) described in S6 Table, extreme low parameters (in blue) or extreme high parameters (in yellow) of *α*_*u*_ (leftmost graph), *α*_*v*_ (middle left graph), *β*_*u*_ (middle right graph), or *ω* (rightmost graph), as a function of modes **k** (see S1 Text). No patterns form with low values of *α*_*u*_ or *ω* or high values of *α*_*v*_ or *β*_*u*_. (B) Simulations of the unified model with initial conditions corresponding to the Japanese quail and various production rates of the activator (or repressor) by the cells *α*_*u*_ (*α*_*v*_), the saturation threshold *β*_*u*_, and autocatalysis sensitivity *ω* of the activator (other parameters equal, reference parameters are *α*_*u*_ ref = 100, *α*_*v*_ ref = 4,500, *β*_*u*_ ref = 6, and *ω* ref = 40) produce dots varying in size and spacing but in a row-by-row sequence. Ts, simulation time.
(TIF)

S9 Fig Simulations with variation of the *δ*_*u*_, *δ*_*v*_ parameters. (A) Dispersion relations of the unified model with references parameters (‘ref’, in red) described in S6 Table, extreme low parameters (in blue) or extreme high parameters (in yellow) of *δ*_*u*_ (left graph) or *δ*_*v*_ (right graph), as a function of modes **k** (see S1 Text). Patterns are formed in all cases. (B) Simulations of the unified model with initial conditions corresponding to the Japanese quail, and various degradation rates of the activator (or repressor) *δ*_*u*_ (*δ*_*v*_), other parameters otherwise unchanged (reference parameters for degradation rates are given by *δ*_*u*_ ref = 15, *δ*_*v*_ ref = 35) produce dots varying in size and spacing but in a row-by-row sequence. Ts, simulation time.
(TIF)

S10 Fig Quantification of sections within and lateral to β-catenin-expressing areas. Left panel: transverse section of a Japanese quail embryo at stage 0 (corresponding to DAPI-stained picture shown in Fig 5C; upper left panel) shows the location of *β-catenin-*expressing areas. Right panel: black lines on high magnification pictures corresponding to the dotted square “a” are sections of 120 μm in length along which DAPI^+^ cells were quantified. Scale bars, 100 μm. CF, competence front; dm, dorsal midline; FF, feather field; n, neural tube.
(TIF)

S11 Fig Cell proliferation in the feather field at stage 1. Cell proliferation is revealed by BrdU stains (in green, upper panel) on transverse sections of Japanese quail embryos at E5.5 or emu embryos at E17 (stage 1) also stained with DAPI to reveal cell nuclei (in white, middle panel). Bottom graphs: quantifications of DAPI^+^/BrdU^+^ cells in 10 sections along the mediolateral axis (as shown in Fig 5C, **a**) show that proliferation is homogeneous just prior to follicle individualisation in the Japanese quail (BrdU^+^ cells represent on average 20.3% of all DAPI^+^ cells; Friedman test, *p* = 0.26; *n* = 8). In the emu, the proliferation rate is comparatively lower (BrdU^+^ cells represent on average 15.4% of all DAPI^+^ cells; *n* = 2; S3 Data). BrdU, 5-Bromo-2′-deoxyuridine; CF, competence front; dm, dorsal midline; E, embryonic day; FF, feather field; n, neural tube.
(TIF)

S12 Fig Effect of colchicine on overall cell proliferation rate. The rate of cell proliferation as quantified by the proportion of BrdU^+^/DAPI^+^ cells in control or colchicine-treated skin explants is statistically different between regions of 150 μm^2^ in size (shown on an untreated, control skin at early stage 2; left panel), located medially to the first-formed row (green square and left graphs; Student *t* tests, *p* = 0.0002), in the first-formed row (blue square and middle graphs, *p* = 0.001), and laterally to the first-formed row (red square and right graphs, *p* < 0.0001; S4 Data). BrdU, 5-Bromo-2′-deoxyuridine.
(TIF)

S13 Fig Visualisation of cell nuclei with DAPI stains on skin explants. DAPI stains on control skin explants of Japanese quail embryos prepared at E6 and fixed at early, intermediate, and late stage 2 (i.e., during the formation of fr#3) and corresponding to BrdU stains shown in Fig 6C (upper panels) reveal the nuclei of all skin cells. BrdU, 5-Bromo-2′-deoxyuridine; E, embryonic day; fr, feather row.
(TIF)

S1 Table Number of embryos assessed for β-catenin expression. (DOCX)

S2 Table Tested doses of colchicine. (DOCX)

S3 Table In vivo measurements and in silico domain units. (DOCX)

S4 Table Parameters of initial conditions of simulation. (DOCX)

S5 Table Tested reaction–diffusion models. (DOCX)

S6 Table Reference parameters of the unified model. (DOCX)

S1 Text Stability analysis of the unified model. (PDF)

S1 Data Data pertaining to Fig 5C. (XLSX)

S2 Data Data pertaining to Fig 6C. (XLSX)

S3 Data Data pertaining to S11 Fig. (XLSX)

S4 Data Data pertaining to S12 Fig. (XLSX)

S5 Data Data pertaining to S3 Table. (XLSX)

## Abbreviations

BMP: bone morphogenetic protein
BrdU: 5-Bromo-2′-deoxyuridine
CF: competence front
dr: dotted row
E: embryonic day
FGF: fibroblast growth factor
ns: nonsignificant
