# Supplementary figures and images for: Symmetry breaking in the embryonic skin triggers directional and sequential plumage patterning
Source: PLoS Biol. 2019 Oct 2;17(10):e3000448. doi: 10.1371/journal.pbio.3000448 (PMC6791559; doi:10.1371/journal.pbio.3000448)

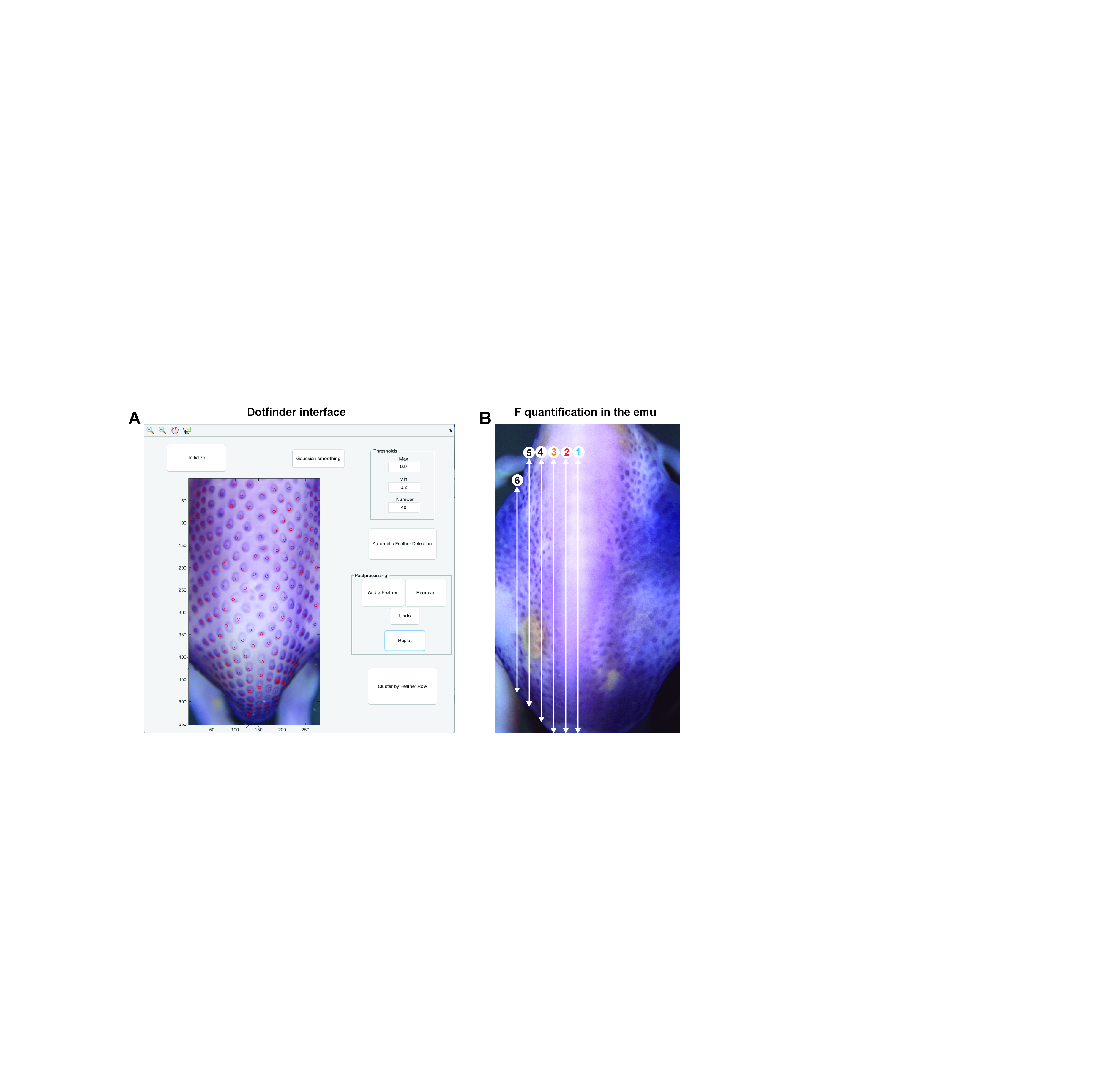

Supplement: S1 Fig — (A) User interface for the custom Matlab program (Dotfinder) for automatic quantification of feather follicles in pictures of whole embryos stained with β-catenin (a Japanese quail at E7.5 is shown), flat skins, and dots in simulation results. The software allows threshold adjustment of the image, automatic detection, postprocessing of false positives and negatives, and clustering of follicle/dot rows. (B) In the emu, the mean number of feathers per row (F) (i.e., comparable to fr#1–6 in other species) was counted along virtual, equally spaced lines extending from wings to tail (here, at E17). E, embryonic day; fr, feather row. (TIF) [file pbio.3000448.s001.tif]

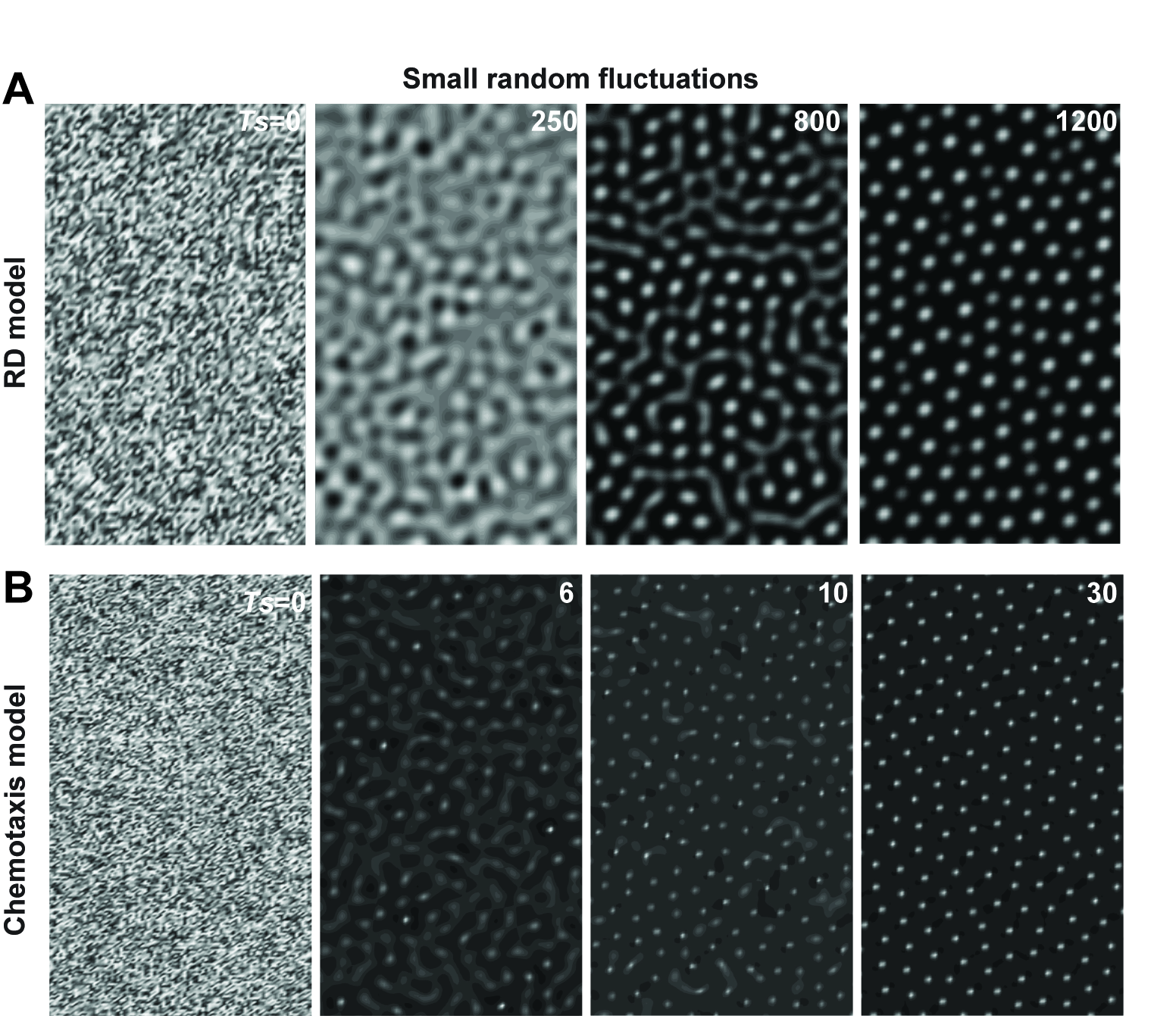

Supplement: S2 Fig — Simulations of a (A) reaction–diffusion model [15] or (B) chemotaxis model [29] produce dotted patterns when initiated on small random fluctuations. Ts, simulation time. (TIF) [file pbio.3000448.s002.tif]

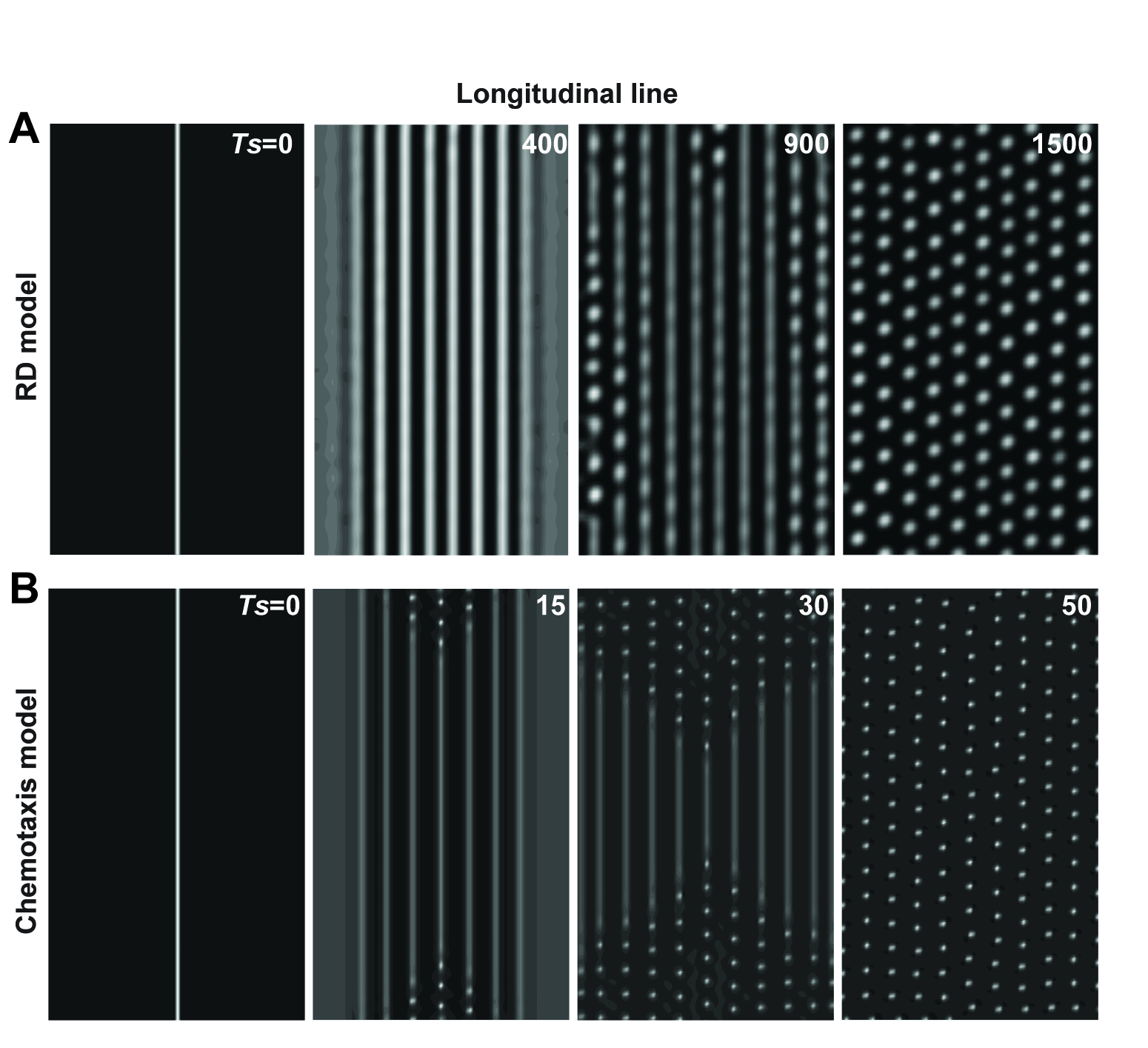

Supplement: S3 Fig — (A) When a reaction–diffusion model [15] is forced onto an initial longitudinal line, it produces stripes that divide into dots to create a dotted motif. (B) When a chemotaxis model [29] is similarly simulated in the same conditions, it produces stripes that simultaneously that divide into dots, starting in the anterior and posterior regions first and travelling towards the centre, resulting in a dotted motif model. Ts, simulation time. (TIF) [file pbio.3000448.s003.tif]

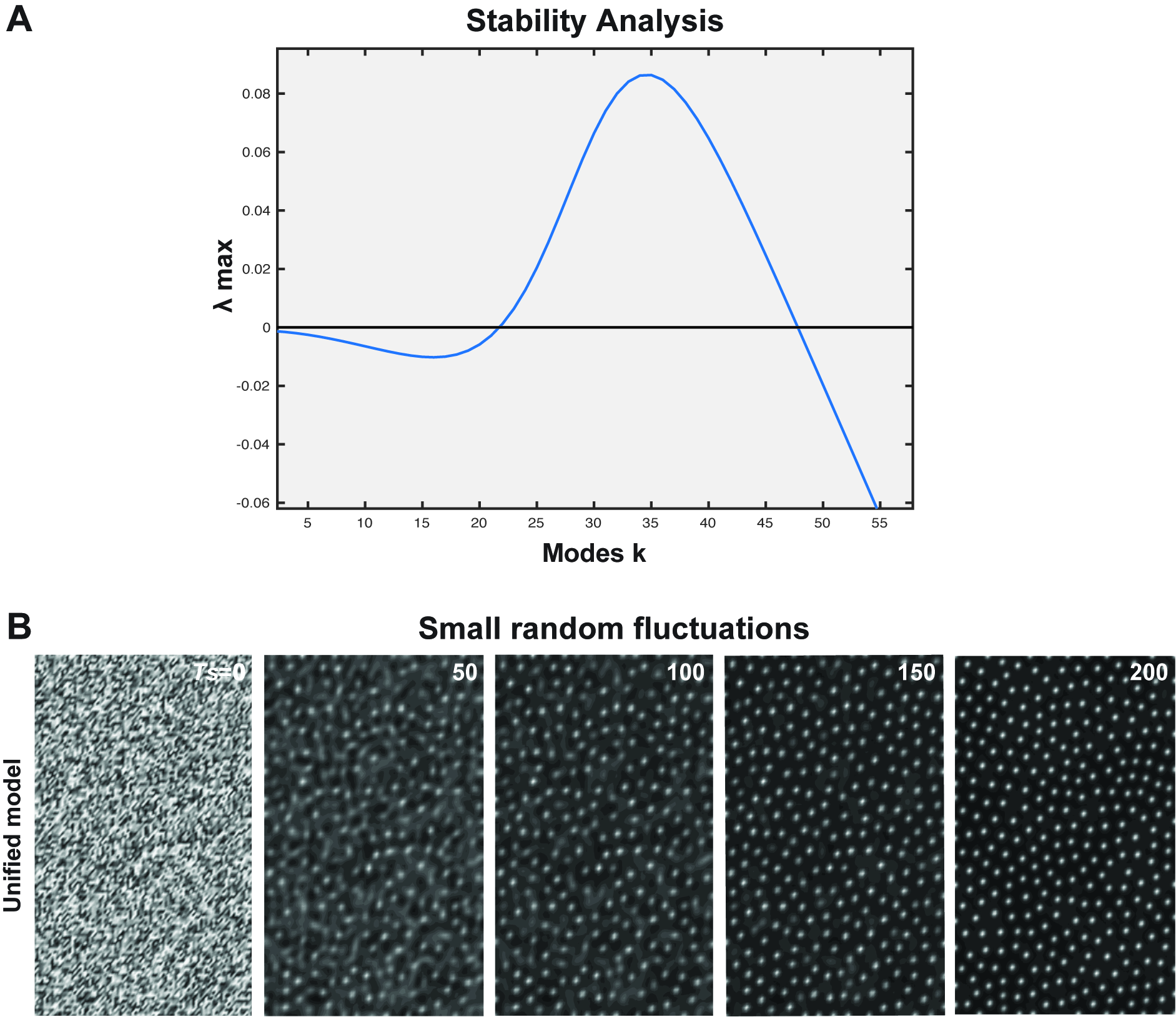

Supplement: S4 Fig — (A) Dispersion relation of the unified model with parameters described in S6 Table and as a function of possible modes of instability k (see S1 Text). The curve attains values above 0, showing that the unified model has Turing instability: nonstable modes amplify from small perturbations and can produce patterns with a periodicity related to these modes. (B) Simulations of the unified model (with parameters described in S6 Table) produce dots that appear simultaneously across the whole frame when initiated on small random fluctuations. Ts, simulation time. (TIF) [file pbio.3000448.s004.tif]

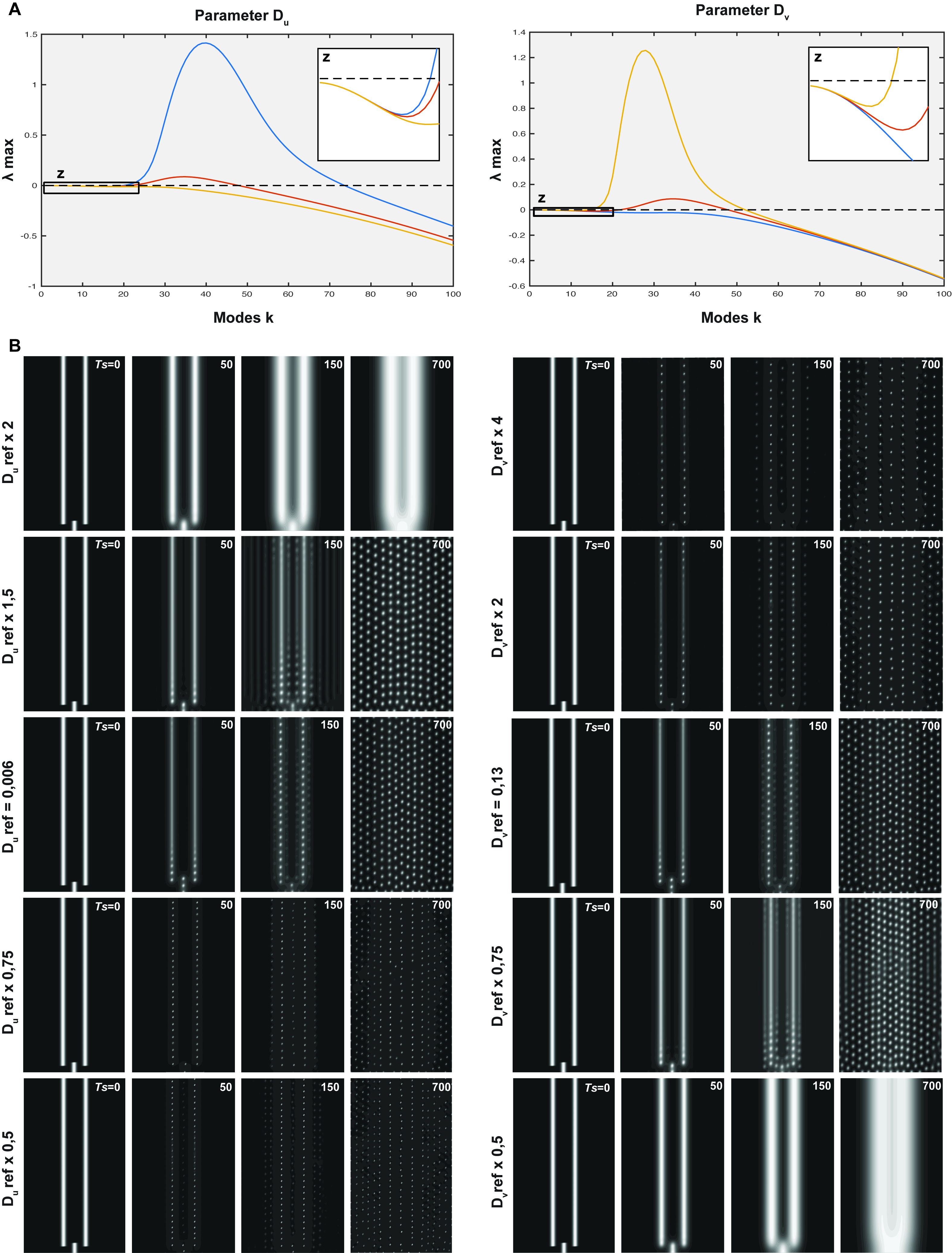

Supplement: S6 Fig — (A) Dispersion relation of the unified model with reference parameters (in red [13]) described in S6 Table, extreme low diffusion parameters (in blue) or extreme high diffusion parameters (in yellow), for Du (left graph) or Dv (middle graph), as a function of possible modes of instability k (see S1 Text). No patterns form when the activator diffusion Du is too high or the inhibitor diffusion Dv is too low. Right graph: depending on values of combined Du and Dv, theoretically derived pattern formation (in white) or homogeneous solutions (in black) occur. (B) Simulations of the unified model with initial conditions corresponding to the Japanese quail, and various diffusivity of the activator Du (left panels), of the inhibitor Dv (right panels), other parameters otherwise equal (reference parameters are Du reference = 0.006 and Dv reference = 0.13), produce dots varying in size and spacing but in a row-by-row sequence. Right panels: simulations of the unified model at the equilibrium are consistent with pattern formation when both Du and Dv vary (i.e., for 9 choices of diffusions; red stars). Ts, simulation time. (TIF) [file pbio.3000448.s006.tif]

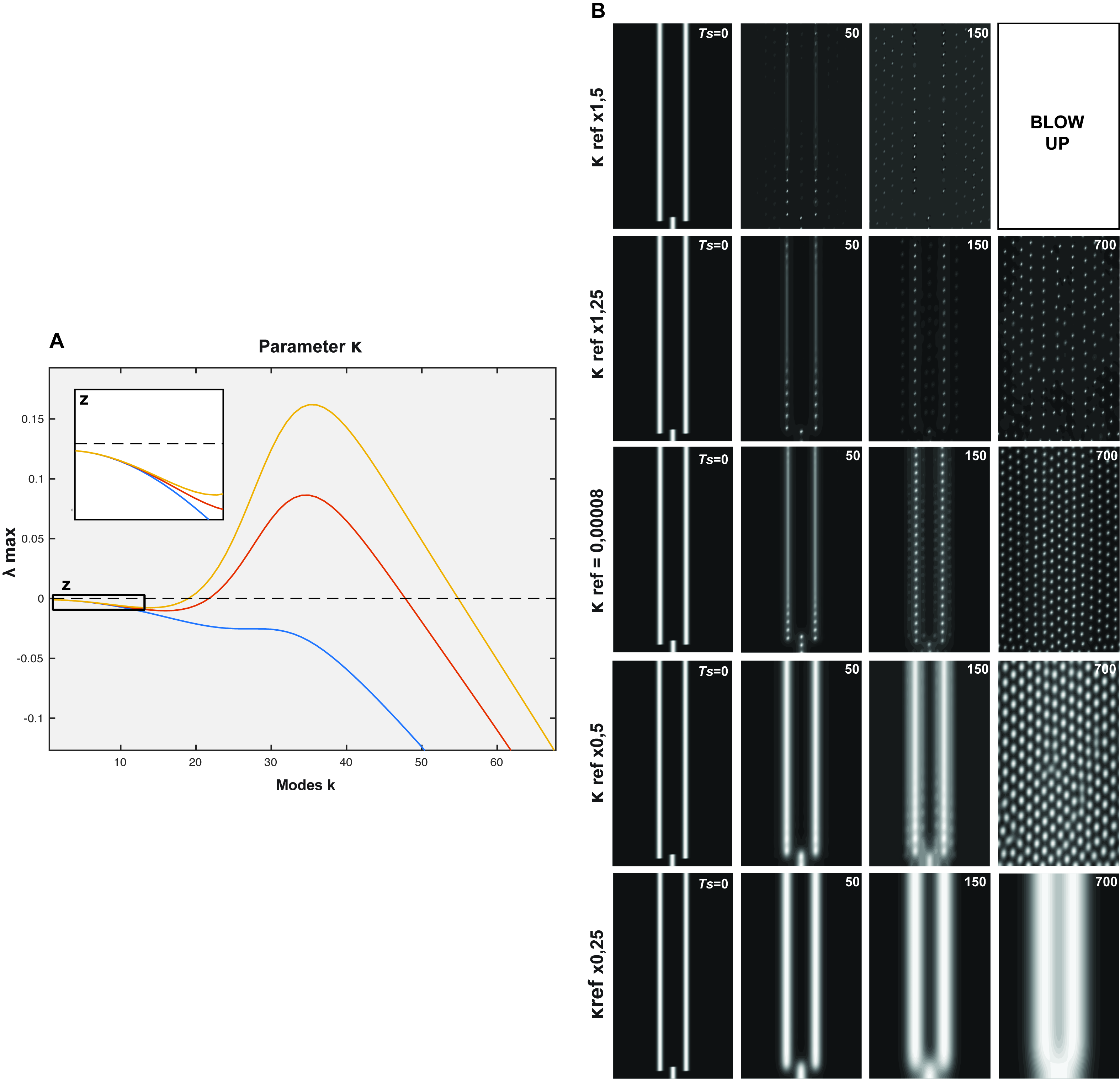

Supplement: S7 Fig — (A) Left graph: dispersion relations of the unified model with reference parameters (‘ref’, in red) described in S6 Table, extreme low parameters (in blue) or extreme high parameters (in yellow) of κ as a function of modes k (see S1 Text). No patterns form with extreme low κ parameters, but patterns occur with high κ. Right graph: depending on values of combined Du and κ, pattern formation occurs (in white) or not (in black). (B) Left panels: simulations of the unified model with initial conditions corresponding to the Japanese quail, and various diffusivity of the sensitivity to chemotaxis κ (other parameters equal, references parameters are κ ref = 0.00008) produce dots varying in size and spacing but in a row-by-row sequence. Right panels: simulations of the unified model at the equilibrium are consistent with pattern formation when both Du and κ vary (i.e., for 9 choices of diffusions; red stars). Ts, simulation time. (TIF) [file pbio.3000448.s007.tif]

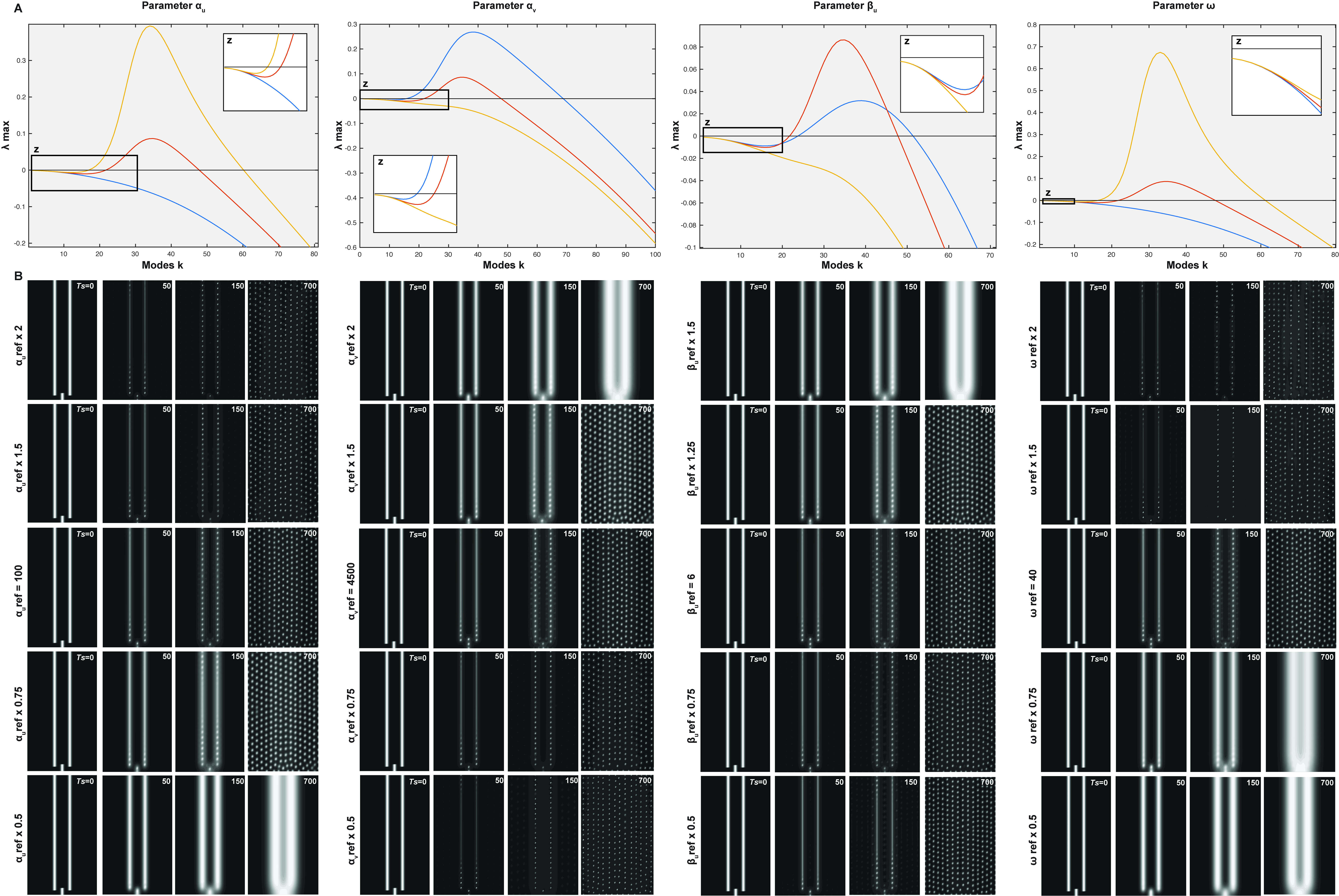

Supplement: S8 Fig — (A) Dispersion relations of the unified model with references parameters (‘ref’, in red) described in S6 Table, extreme low parameters (in blue) or extreme high parameters (in yellow) of αu (leftmost graph), αv (middle left graph), βu (middle right graph), or ω (rightmost graph), as a function of modes k (see S1 Text). No patterns form with low values of αu or ω or high values of αv or βu. (B) Simulations of the unified model with initial conditions corresponding to the Japanese quail and various production rates of the activator (or repressor) by the cells αu (αv), the saturation threshold βu, and autocatalysis sensitivity ω of the activator (other parameters equal, reference parameters are αu ref = 100, αv ref = 4,500, βu ref = 6, and ω ref = 40) produce dots varying in size and spacing but in a row-by-row sequence. Ts, simulation time. (TIF) [file pbio.3000448.s008.tif]

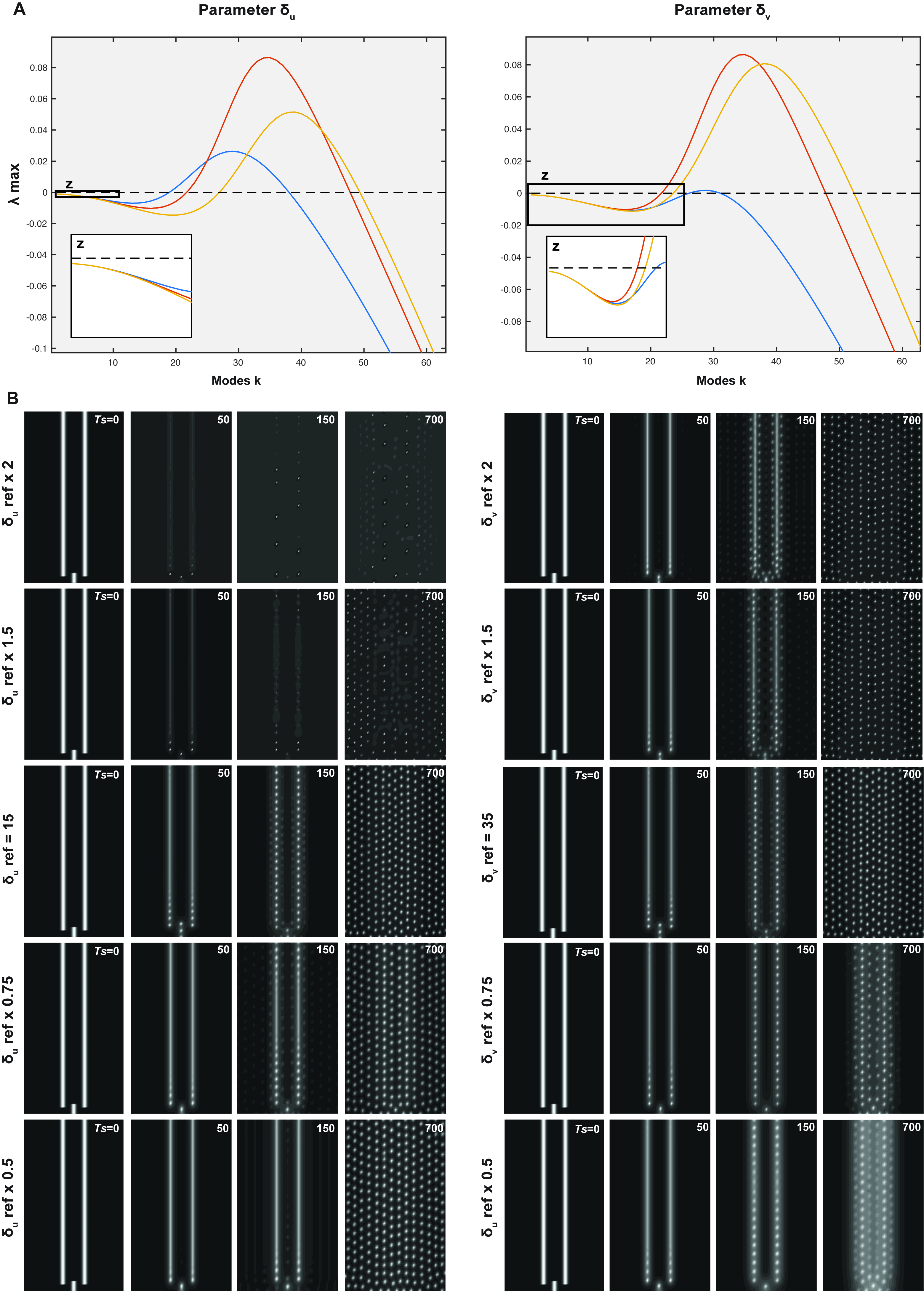

Supplement: S9 Fig — (A) Dispersion relations of the unified model with references parameters (‘ref’, in red) described in S6 Table, extreme low parameters (in blue) or extreme high parameters (in yellow) of δu (left graph) or δv (right graph), as a function of modes k (see S1 Text). Patterns are formed in all cases. (B) Simulations of the unified model with initial conditions corresponding to the Japanese quail, and various degradation rates of the activator (or repressor) δu (δv), other parameters otherwise unchanged (reference parameters for degradation rates are given by δu ref = 15, δv ref = 35) produce dots varying in size and spacing but in a row-by-row sequence. Ts, simulation time. (TIF) [file pbio.3000448.s009.tif]

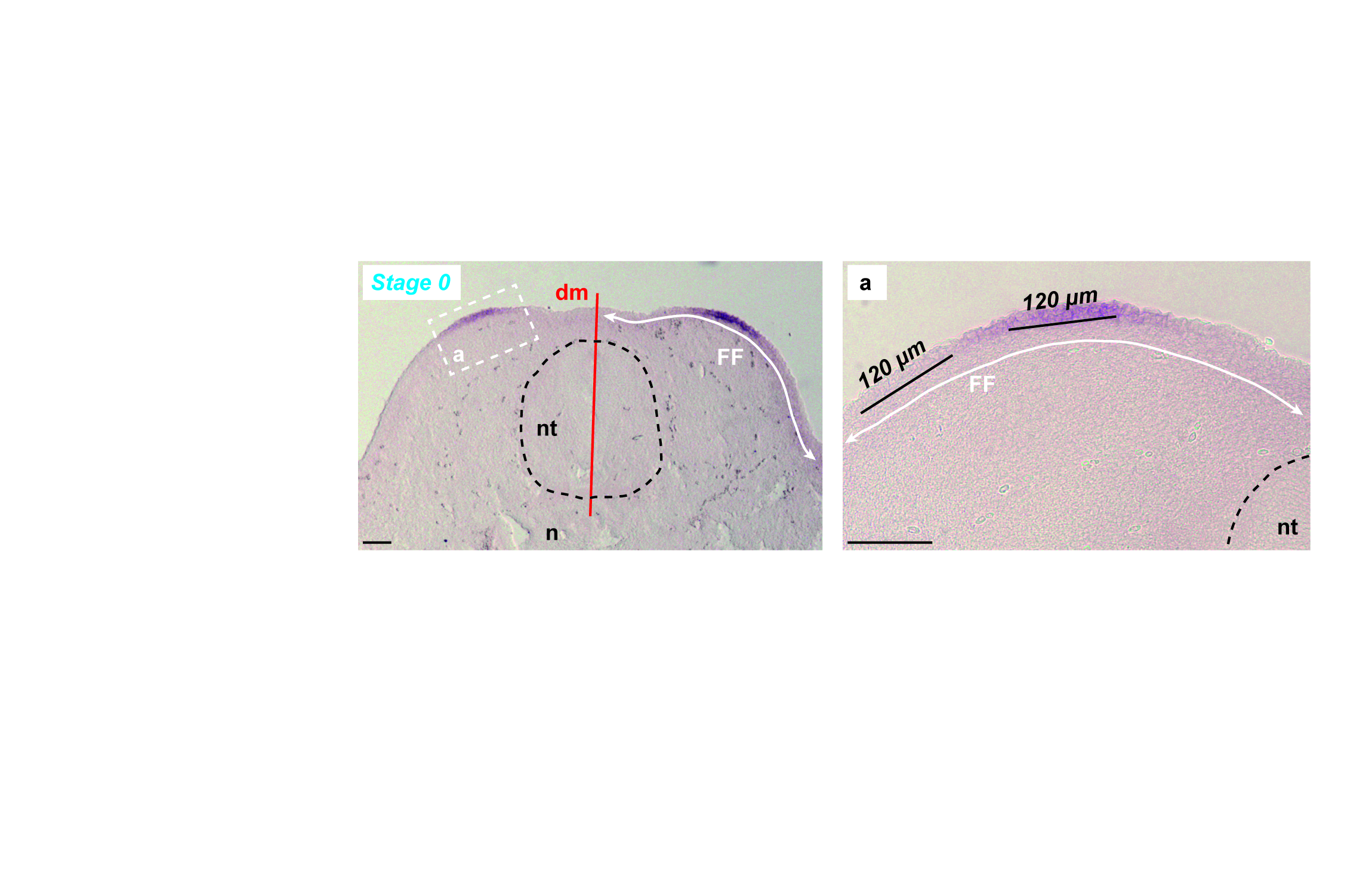

Supplement: S10 Fig — Left panel: transverse section of a Japanese quail embryo at stage 0 (corresponding to DAPI-stained picture shown in Fig 5C; upper left panel) shows the location of β-catenin-expressing areas. Right panel: black lines on high magnification pictures corresponding to the dotted square “a” are sections of 120 μm in length along which DAPI+ cells were quantified. Scale bars, 100 μm. CF, competence front; dm, dorsal midline; FF, feather field; n, neural tube. (TIF) [file pbio.3000448.s010.tif]

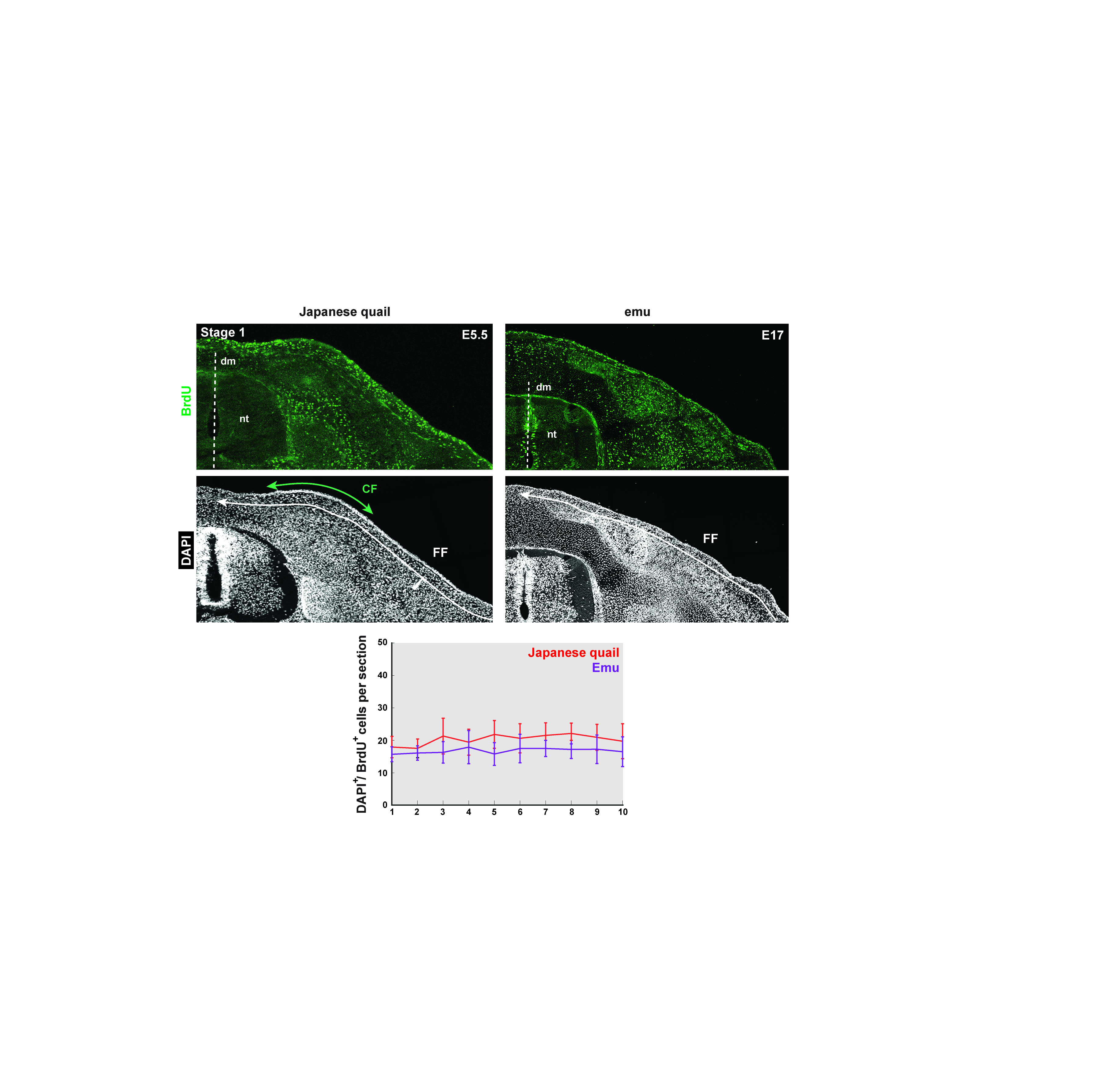

Supplement: S11 Fig — Cell proliferation is revealed by BrdU stains (in green, upper panel) on transverse sections of Japanese quail embryos at E5.5 or emu embryos at E17 (stage 1) also stained with DAPI to reveal cell nuclei (in white, middle panel). Bottom graphs: quantifications of DAPI+/BrdU+ cells in 10 sections along the mediolateral axis (as shown in Fig 5C, a) show that proliferation is homogeneous just prior to follicle individualisation in the Japanese quail (BrdU+ cells represent on average 20.3% of all DAPI+ cells; Friedman test, p = 0.26; n = 8). In the emu, the proliferation rate is comparatively lower (BrdU+ cells represent on average 15.4% of all DAPI+ cells; n = 2; S3 Data). BrdU, 5-Bromo-2′-deoxyuridine; CF, competence front; dm, dorsal midline; E, embryonic day; FF, feather field; n, neural tube. (TIF) [file pbio.3000448.s011.tif]

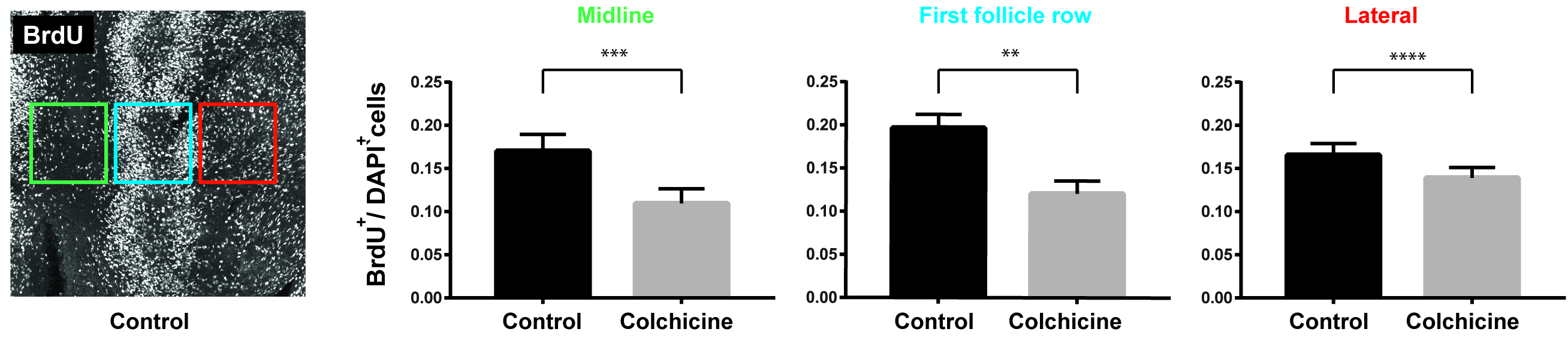

Supplement: S12 Fig — The rate of cell proliferation as quantified by the proportion of BrdU+/DAPI+ cells in control or colchicine-treated skin explants is statistically different between regions of 150 μm2 in size (shown on an untreated, control skin at early stage 2; left panel), located medially to the first-formed row (green square and left graphs; Student t tests, p = 0.0002), in the first-formed row (blue square and middle graphs, p = 0.001), and laterally to the first-formed row (red square and right graphs, p < 0.0001; S4 Data). BrdU, 5-Bromo-2′-deoxyuridine. (TIF) [file pbio.3000448.s012.tif]

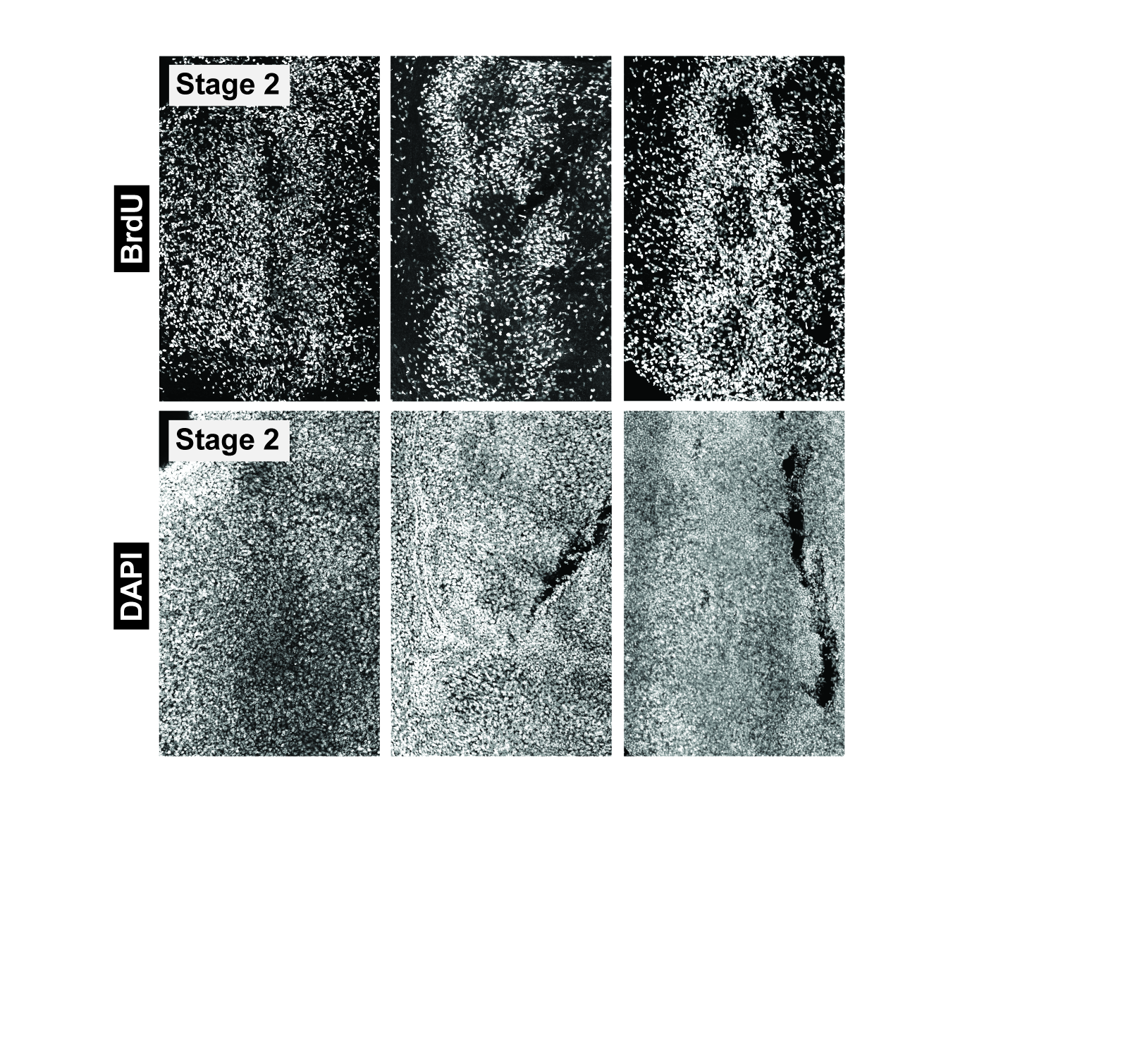

Supplement: S13 Fig — DAPI stains on control skin explants of Japanese quail embryos prepared at E6 and fixed at early, intermediate, and late stage 2 (i.e., during the formation of fr#3) and corresponding to BrdU stains shown in Fig 6C (upper panels) reveal the nuclei of all skin cells. BrdU, 5-Bromo-2′-deoxyuridine; E, embryonic day; fr, feather row. (TIF) [file pbio.3000448.s013.tif]
