# Supplementary material for: Symmetry breaking in the embryonic skin triggers directional and sequential plumage patterning
Source: PLoS Biol. 2019 Oct 2;17(10):e3000448. doi: 10.1371/journal.pbio.3000448 (PMC6791559; doi:10.1371/journal.pbio.3000448)
Supplement: S1 Table — (DOCX) [file pbio.3000448.s014.docx]

**S1 Table: Number of embryos assessed for *ß-catenin* expression**

| **Stage (E)** | Stage 1 | Stage 2 | Stage 3 | Stage 4 | Stage 5 |
| --- | --- | --- | --- | --- | --- |
| *Gallus gallus* | 2 | 3 | 3 | 4 | 6 |
| *Coturnix japonica* | 5 | 4 | 2 | 4 | 4 |
| *Phasianus colchicus* | 5 | 3 | 3 | 4 | 5 |
| *Dromaius novaehollandiae* | 2 | 1 | 1 | 1 | 1 |
| *Taeniopygia guttata* | 4 | 4 | 6 | 7 | 9 |
| *Pygoscelis papua* | 2 | 2 | 1 | 2 | 2 |
