# Supplementary material for: Symmetry breaking in the embryonic skin triggers directional and sequential plumage patterning
Source: PLoS Biol. 2019 Oct 2;17(10):e3000448. doi: 10.1371/journal.pbio.3000448 (PMC6791559; doi:10.1371/journal.pbio.3000448)
Supplement: S2 Table — (DOCX) [file pbio.3000448.s015.docx]

**S2 Table: *In vivo* measurements and *in silico* domain units**

|  | *G. gallus* | *C. japonica* | | *P. colchicus* | | *T. guttata* | | *D. novaehollandiae* | |
| --- | --- | --- | --- | --- | --- | --- | --- | --- | --- |
| Wings-tail distance L (mm) | 11 | | 10 | | 10.1 | | 6.8 | | 13.5 |
| Wing width l (mm) | 5 | | 4.7 | | 4.8 | | 2.5 | | 7 |
| *In silico* domain size L_s_ x l_s_ | 5 x 3.5 | | 4.5 x 3.3 | | 4.6 x 3.4 | | 3.1 x 1.8 | | 6.1 x 4.9 |
