# Supplementary material for: Symmetry breaking in the embryonic skin triggers directional and sequential plumage patterning
Source: PLoS Biol. 2019 Oct 2;17(10):e3000448. doi: 10.1371/journal.pbio.3000448 (PMC6791559; doi:10.1371/journal.pbio.3000448)
Supplement: S3 Table — (DOCX) [file pbio.3000448.s016.docx]

**S3 Table: Parameters of initial conditions of simulation**

| **Parameters** | *G. gallus* | *C. japonica* | *P. colchicus* | *T. guttata* |
| --- | --- | --- | --- | --- |
| m | 0.5 | | | |
| a | 2 | | | |
| $s_{m}$ | 200 | 200 | 20 | 400 |
| $s_{l}$ | 200 | 200 | 200 | 400 |
| $x_{l}$ | 0.25 | 0.36 | 0.25 | 0.2 |
| $y_{l}$ | 2.6 | 0.1 | 0.1 | 0.9 |
| $y_{m}$ | 2.8 | 0.3 | 2 | 0.85 |
