# Supplementary material for: Symmetry breaking in the embryonic skin triggers directional and sequential plumage patterning
Source: PLoS Biol. 2019 Oct 2;17(10):e3000448. doi: 10.1371/journal.pbio.3000448 (PMC6791559; doi:10.1371/journal.pbio.3000448)
Supplement: S4 Table — (DOCX) [file pbio.3000448.s017.docx]

**S4 Table: Tested reaction-diffusion models**

| $\left\{ \begin{aligned} \partial_{t}u= D_{u}\Delta u+\min\left( \max\left( a_{u}u+b_{u}v+c_{u},0 \right),F_{max} \right) \\ \partial_{t}v=D_{v}\Delta v+\min\left( \max\left( a_{v}u+b_{v}v+c_{v},0 \right),G_{max} \right) \end{aligned} \right.$ |
| --- |
| $\left\{ \begin{aligned} \partial_{t}u= D_{u}\Delta u+p_{u}\frac{u^{2}}{(1+k_{2}v)({k_{1}}^{2}+u^{2})}+\alpha_{u}-\delta_{u}u \\ \partial_{t}v=D_{v}\Delta v+ p_{v}\frac{u^{2}}{{k_{3}}^{2}+u^{2}}+\alpha_{v}-\delta_{v}v \end{aligned} \right.$ |
| $\left\{ \begin{aligned} \partial_{t}u= D_{u}\Delta u+p_{u}\frac{u^{2}}{(\gamma+v)(1+ku^{2})}-\delta_{u}u \\ \partial_{t}v=D_{v}\Delta v+ p_{v}\frac{u^{2}}{(\gamma+v)(1+ku^{2})}-\delta_{v}v \end{aligned}\boldsymbol{.} \right.$ |
| $\left\{ \begin{aligned} \partial_{t}u= D_{u}\Delta u-uv^{2}+\alpha_{u}-\delta_{u}u \\ \partial_{t}v=D_{v}\Delta v+uv^{2}-\delta_{v}v \end{aligned}\boldsymbol{.} \right.$ |
| $\left\{ \begin{aligned} \partial_{t}u= D_{u}\Delta u-\frac{4uv}{1+v^{2}}+\alpha_{u}-u \\ \partial_{t}v=D_{v}\Delta v+ \alpha_{v}(u-\frac{uv}{1+v^{2}}) \end{aligned}\boldsymbol{.} \right.$ |
