# Supplementary material for: Symmetry breaking in the embryonic skin triggers directional and sequential plumage patterning
Source: PLoS Biol. 2019 Oct 2;17(10):e3000448. doi: 10.1371/journal.pbio.3000448 (PMC6791559; doi:10.1371/journal.pbio.3000448)
Supplement: S5 Table — (DOCX) [file pbio.3000448.s018.docx]

**S5 Table: Reference parameters of the unified model**

| Parameter | **Value** | **Biological interpretation** |
| --- | --- | --- |
| D_n_ | 7*10^-5^ | Cell diffusion rate |
| D_u_ | 6*10^-3^ | Activator’s diffusion rate |
| D_v_ | 0.13 | Inhibitor diffusion rate |
| $\kappa$ | 8*10^-5^ | Chemotaxis sensitivity |
| $\delta_{u}$ | 15 | Activator degradation rate |
| $\delta_{v}$ | 35 | Inhibitor degradation rate |
| $\alpha$ _u_ | 100 | Activator production rate by cells |
| $\omega$ | 40 | Activator autocatalysis sensitivity |
| $\beta$ _u_ | 6 | Activator saturation threshold |
| $\alpha$ _v_ | 4500 | Inhibitor production rate by cells |
| $\alpha$ | 1*10^-3^ | Proliferation rate |
| $\beta$ | 3 | Cell density threshold |
