# Supplementary material for: Symmetry breaking in the embryonic skin triggers directional and sequential plumage patterning
Source: PLoS Biol. 2019 Oct 2;17(10):e3000448. doi: 10.1371/journal.pbio.3000448 (PMC6791559; doi:10.1371/journal.pbio.3000448)
Supplement: S6 Table — (DOCX) [file pbio.3000448.s019.docx]

**S6 Table: Tested doses of Colchicine**

| Colchicine concentration (mg/L) | 0.001 | 0.01 | 0.025 | 0.05 | 0.1 | 0.2 | 0.5 | 1 | 2 | 4 | 40 |
| --- | --- | --- | --- | --- | --- | --- | --- | --- | --- | --- | --- |
| Lethality | no | no | no | no | yes | yes | yes | yes | yes | yes | yes |
