# Supplementary material for: Symmetry breaking in the embryonic skin triggers directional and sequential plumage patterning
Source: PLoS Biol. 2019 Oct 2;17(10):e3000448. doi: 10.1371/journal.pbio.3000448 (PMC6791559; doi:10.1371/journal.pbio.3000448)
Supplement: S1 Text — (PDF) [file pbio.3000448.s020.pdf]

In this supplement we analyze the stability of spatially homogeneous solutions, and determine Turing instabilities associated with the formation of patterns on bird skin for our unified model:

$$\begin{cases}\partial_t n = e(n,u,v) = D_n \Delta n - \nabla.(\kappa n \nabla u) + \alpha_n n \left(1 - \frac{n}{\beta_n}\right) \\ \partial_t u = f(n,u,v) = D_u \Delta u + \frac{\alpha_u n (1+\omega u^2)}{(\beta_u + u^2)(1+v)} - \delta_u u \\ \partial_t v = g(n,u,v) = D_v \Delta v + \alpha_v n u^2 - \delta_v v \end{cases} \quad (1)$$

Homogeneous steady states

Spatially homogeneous equilibria are solutions of the equation (1) $(n(t,r), u(t,r), v(t,r))$ that are independent of time (steady state, or equilibrium) and of the position r on the tissue (spatially homogeneous). These spatially homogeneous solutions are thus solutions of the system of algebraic equations:

$$\begin{cases}\alpha_n n \left(1 - \dfrac{n}{\beta_n}\right) = 0 \\ \dfrac{\alpha_u n (1+\omega u^2)}{(\beta_u + u^2)(1+v)} - \delta_u u = 0 \\ \alpha_v n u^2 - \delta_v v = 0 \end{cases} \quad (2)$$

that has two relevant solutions:

- the trivial state $(0,0,0)$, corresponding to an unrealistic situation of complete absence of cells in the tissue ($n = 0$), in turn leading to an absence of activator and inhibitor $u = v = 0$. Because of the proliferation term, we expect this solution to be always unstable: a solution of equation (1) starting with a small number of cells will be able to proliferate at a rate equal to α_n (while n is small compared to the tissue capacity) and thus the system will move away from that steady state.
- A non-trivial equilibrium $(\mathbf{n_s}, \mathbf{u_s}, \mathbf{v_s})$ where the number of cells reaches the carrying capacity of the tissue $\mathbf{n_s} = \beta_n$, and $\mathbf{v_s} = \frac{\alpha_v \beta_n}{\delta_v} \mathbf{u_s}^2$ and $\mathbf{u_s}$ is a solution to the degree five polynomial:

  $$-\frac{\delta_u \alpha_v \beta_n}{\delta_v} \mathbf{u_s}^5 - \left(\delta_u + \frac{\delta_u \alpha_v \beta_u \beta_n}{\delta_v}\right) \mathbf{u_s}^3 + \alpha_u \beta_n \omega \mathbf{u_s}^2 - \delta_u \beta_u \mathbf{u_s} + \alpha_u \beta_n = 0.$$

  This degree-five polynomial can have up to 5 solutions. However, we found numerically that only one solution is real, and the other four are formed of two pairs of complex conjugated numbers. Only the real solution is relevant for our study.

Linear Stability - General Analysis

To assess stability of the equilibria found, we consider whether small perturbations about one of solution are initially damped or amplified. When small perturbations vanish, the equilibrium is said to be stable, and it is otherwise unstable.

Consider a small initial perturbation of the equilibrium of size ε, and let us denote the solution $n = \mathbf{n_0} + \varepsilon \phi_n$, $u = \mathbf{u_0} + \varepsilon \phi_u$ and $v = \mathbf{v_0} + \varepsilon \phi_v$ for some functions (ϕ_n, ϕ_s, ϕ_v) to be determined and $(\mathbf{n_0}, \mathbf{u_0}, \mathbf{v_0})$ one of the spatially homogeneous solutions computed above. Using the fact that n is solution to the equation, we obtain:

$$\varepsilon \partial_t \phi_n = \varepsilon D_n \Delta \phi_n - \varepsilon \nabla.(\kappa \phi_n \nabla \mathbf{u_0}) - \varepsilon \nabla.(\kappa \mathbf{n_0} \nabla \phi_u) - \varepsilon^2 \nabla.(\kappa \phi_n \nabla \phi_u) + \varepsilon \alpha_n \left(1 - \frac{2\mathbf{n_0}}{\beta_n}\right) \phi_n + \varepsilon^2 \alpha_n \frac{\phi_n^2}{\beta_n} \quad (3)$$

and using the fact that $\mathbf{u_0}$ is spatially homogeneous (so the gradient is 0), that $\mathbf{n_0}$ is spatially homogeneous (thus treated as a constant for the divergence in the chemotaxis term) and neglecting terms of order ε^2 (and possibly higher), one obtains the linearized equation on ϕ_n:

$$\partial_t \phi_n = D_n \Delta \phi_n - \kappa \mathbf{n_0} \Delta \phi_u + \alpha_n \left(1 - \frac{2\mathbf{n_0}}{\beta_n}\right) \phi_n. \tag{4}$$

Proceeding in the same fashion for the other two variables, we obtain the linearized system:

$$\begin{cases} \partial_t \phi_n &= D_n \Delta \phi_n - \kappa \mathbf{n_0} \Delta \phi_u + \alpha_n \left(1 - \frac{2\mathbf{n_0}}{\beta_n}\right) \phi_n \\ \partial_t \phi_u &= D_u \Delta \phi_u + \frac{\alpha_u (1+\omega \mathbf{u_0}^2)}{(\beta_u + \mathbf{u_0}^2)(1+\mathbf{v_0})} \phi_n + \left(\frac{2\alpha_u \mathbf{n_0} \mathbf{u_0} (\omega \beta_u - 1)}{(\beta_u + \mathbf{u_0}^2)^2 (1+\mathbf{v_0})} - \delta_u \right) \phi_u - \frac{\alpha_u \mathbf{n_0} (1+\omega \mathbf{u_0}^2)}{(\beta_u + \mathbf{u_0}^2)(1+\mathbf{v_0})^2} \phi_v \\ \partial_t \phi_v &= D_v \Delta \phi_v + \alpha_v \mathbf{u_0}^2 \phi_n + 2\alpha_v \mathbf{n_0} \mathbf{u_0} \phi_u - \delta_v \phi_v \end{cases} \tag{5}$$

The solution $(\mathbf{n_0}, \mathbf{u_0}, \mathbf{v_0})$ is stable if solutions (ϕ_n, ϕ_u, ϕ_v) of the linear perturbation equation decays to zero in time. To this purpose, we will decompose the initial shape of the perturbation on the Fourier basis, which is given by the functions $W_{k_1,k_2} = \cos \frac{k_1 \pi x}{L_1} \cos \frac{k_2 \pi y}{L_2}$ to ensure that our boundary conditions are satisfied. Mathematically, this set of functions is said to form a Hilbert basis (in the $\mathbb{L}^2$ sense) of the set of functions Ω such that $\partial_x f(0,0) = \partial_x f(L_1, 0) = \partial_y f(0,0) = \partial y f(0, L_2) = 0$, and any initial perturbation can be decomposed univocally in this Fourier basis:

$$\phi_z(x, y, t=0) = \sum_{k_1=1}^{\infty} \sum_{k_2=1}^{\infty} C_z^{k_1,k_2} \cos \frac{k_1 \pi x}{L_1} \cos \frac{k_2 \pi y}{L_2}.$$

Stability analysis thus amounts to investigating whether Fourier modes are amplified and damped. If at least one Fourier mode is amplified, the solution is unstable. The choice of decomposing the initial condition on the Fourier basis is relevant to study equation (5) because Fourier modes are eigenfunctions of the diffusion operator:

$$\Delta W_{k_1,k_2} = \mu_{k_1,k_2} W_{k_1,k_2}$$

associated with the eigenvalues:

$$\mu_{k_1,k_2} = -\pi^2 \left(\frac{k_1^2}{L_1^2} + \frac{k_2^2}{L_2^2} \right)$$

Moreover, because the equation (5) is linear, from the principle of superposition of solutions for linear equations, it is sufficient to consider the evolution of a single mode. To assess whether solutions are damped or amplified, we thus look for solutions ϕ_z (for $z = n, u, v$) of the form[1]

$$\phi_z(x, y, t) = e^{\lambda t} C_z \, W_{k_1,k_2}(x, y) \tag{6}$$

for C_z some constant and λ a complex number whose real part is the rate at which the perturbation is damped (real part of $\lambda < 0$) or amplified (real part of $\lambda > 0$). We note $\mathbf{\Phi} = (\phi_n, \phi_u, \phi_v)$ and $\mathbf{C} = (C_n, C_u, C_v)$. In this equation, we observed that if a mode is amplified on one coordinate, it is

[1] Rigorously, one may choose a distinct Fourier mode on each coefficient, yielding an equation with 6 unknown. In practice, when an instability occurs yielding a pattern in this system, all coordinates display a similar periodicity, and it is thus relevant to consider instability when the initial condition on n, u, v is perturbed by the same Fourier mode.

also on the other, and we thus make the simplifying assumption $k_1^n = k_1^u = k_1^v$ and $k_2^n = k_2^u = k_2^v$. The linearized equation for this perturbation reads:

$$\frac{\partial \mathbf{\Phi}}{\partial t} = \begin{pmatrix} D_n\mu_{k_1,k_2} + \alpha_n\left(1 - \frac{2\mathbf{n_0}}{\beta_n}\right) & -\kappa\mathbf{n_0}\mu_{k_1,k_2} & 0 \\ \frac{\alpha_u\left(1+\omega\mathbf{u_0}^2\right)}{(\beta_u+\mathbf{u_0}^2)(1+\mathbf{v_0})} & D_u\mu_{k_1,k_2} + \frac{2\alpha_u\mathbf{n_0}\mathbf{u_0}(\omega\beta_u-1)}{(\beta_u+\mathbf{u_0}^2)^2(1+\mathbf{v_0})} - \delta_u & -\frac{\alpha_u\mathbf{n_0}\left(1+\omega\mathbf{u_0}^2\right)}{(\beta_u+\mathbf{u_0}^2)(1+\mathbf{v_0})^2} \\ \alpha_v\mathbf{u_0}^2 & 2\alpha_v\mathbf{n_0}\mathbf{u_0} & D_v\mu_{k_1,k_2} - \delta_v \end{pmatrix} \cdot \mathbf{\Phi} \quad (7)$$

$$=: \begin{pmatrix} \tilde{e}_n(k_1,k_2) & \tilde{e}_u(k_1,k_2) & e_v \\ f_n & \tilde{f}_u(k_1,k_2) & f_v \\ g_n & g_u & \tilde{g}_v(k_1,k_2) \end{pmatrix} \cdot \mathbf{\Phi}. \quad (8)$$

implying, when inserting the specific form of $\mathbf{\Phi}$ in the equation, that:

$$\lambda\mathbf{C} = \begin{pmatrix} \tilde{e}_n(k_1,k_2) & \tilde{e}_u(k_1,k_2) & e_v \\ f_n & \tilde{f}_u(k_1,k_2) & f_v \\ g_n & g_u & \tilde{g}_v(k_1,k_2) \end{pmatrix} \cdot \mathbf{C}.$$

In other words, λ is an eigenvalue of the matrix on the righthand side.

Stability of the trivial steady state

For the trivial spatially homogeneous state $(0,0,0)$, the matrix associated with stability is given by:

$$\det\begin{pmatrix} \lambda - D_n\mu_{k_1^n,k_2^n} - \alpha_n & 0 & 0 \\ 0 & \lambda - D_u\mu_{k_1^u,k_2^u} + \delta_u & 0 \\ 0 & 0 & \lambda - D_v\mu_{k_1^v,k_2^v} + \delta_v \end{pmatrix} \quad (9)$$

so the expressions of eigenvalues are straightforward:

$$\begin{aligned} \lambda_1 &= D_n\mu_{k_1^n,k_2^n} + \alpha_n \\ \lambda_2 &= D_u\mu_{k_1^u,k_2^u} - \delta_u \\ \lambda_3 &= D_v\mu_{k_1^v,k_2^v} - \delta_v \end{aligned} \quad .$$

Because μ_{k_1,k_2} is non-positive, λ_2, λ_3 are strictly negative, but because $\alpha_n > 0$, λ_1 is positive for $k_1 = k_2 = 0$. This implies that the trivial state is always unstable: a small cell density will progressively grow because of the logistic growth term.

Stability of the non-trivial equilibrium

To determine the values of λ in the case of the non-trivial equilibrium, we use the classical fact that eigenvalues are the roots of the polynomial:

$$\begin{vmatrix} \lambda - \tilde{e}_n(k_1,k_2) & -\tilde{e}_u(k_1,k_2) & -e_v \\ -f_n & \lambda - \tilde{f}_u(k_1,k_2) & -f_v \\ -g_n & -g_u & \lambda - \tilde{g}_v(k_1,k_2) \end{vmatrix} = \lambda^3 + a_2(k_1,k_2)\lambda^2 + a_1(k_1,k_2)\lambda + a_0(k_1,k_2) \quad (10)$$

where

$$\begin{cases} a_2(k_1,k_2) &= -\tilde{e}_n(k_1,k_2) - \tilde{f}_u(k_1,k_2) - \tilde{g}_v(k_1,k_2) \\ a_1(k_1,k_2) &= \tilde{f}_u(k_1,k_2)\tilde{g}_v(k_1,k_2) - g_u f_v - f_n \tilde{e}_u(k_1,k_2) + \tilde{e}_n(k_1,k_2)\tilde{f}_u(k_1,k_2) + \tilde{e}_n(k_1,k_2)\tilde{g}_v(k_1,k_2) \\ a_0(k_1,k_2) &= -\tilde{e}_n(k_1,k_2)\tilde{f}_u(k_1,k_2)\tilde{g}_v(k_1,k_2) + \tilde{e}_n(k_1,k_2)g_u f_v + f_n \tilde{e}_u(k_1,k_2)\tilde{g}_v(k_1,k_2) - g_n \tilde{e}_u(k_1,k_2) f_v. \end{cases} \tag{11}$$

The Routh-Hurwitz criterion provides three conditions for ensuring that eigenvalues have strictly negative real part (i.e. that the equilibrium is stable). In detail, all eigenvalues have strictly negative real part and only if we have simultaneously $a_0,\ a_2 \geq 0$ and $a_2 a_1 > a_0$. An instability thus arises when one of these conditions is not satisfied, i.e.:

$$a_0(k_1,k_2) < 0$$
or
$$a_2(k_1,k_2) < 0$$
or
$$a_2(k_1,k_2)a_1(k_1,k_2) \leq a_0(k_1,k_2)$$

While these expressions may look complex, they are relatively simple functions of μ_{k_1,k_2}. We found that for a variety of parameters in the vicinity of the central parameter chosen, only the term a_0 can break the condition of stability of Routh-Hurwitz.

We plotted in S4 and S6-9 Figs the corresponding dispersion relations showing, for the reference (red), highest (orange) and lowest (blue) displayed parameter sets, the evolution of the real part of largest eigenvalues of the stability matrix (10) as a function of the wavenumbers k (in these plots, we took $k = k_1 = k_2$, allowing a simple visualisation). These curves highlight the existence of *Turing instabilities* in cases where a finite range of positive λ exist: the associated modes become unstable and are amplified, yielding the emergence of a pattern.

We note that the non-trivial steady state is independent of the diffusion and chemotaxis coefficients. We thus considered its stability as D_u and D_v (respectively, D_u and κ) are varied in combination, as is shown in S6 Fig (respectively, S7 Fig). We found that instabilities require to have D_u relatively small and D_v relatively large (respectively, D_u relatively small and κ relatively large). This is perfectly consistent with the loss of patterning when varying these parameters, as observed in S6-7 Fig.
